# Sorghum Association Panel whole‐genome sequencing establishes cornerstone resource for dissecting genomic diversity

**DOI:** 10.1111/tpj.15853

**Published:** 2022-07-05

**Authors:** J. Lucas Boatwright, Sirjan Sapkota, Hongyu Jin, James C. Schnable, Zachary Brenton, Richard Boyles, Stephen Kresovich

**Affiliations:** ^1^ Department of Plant and Environmental Sciences Clemson University Clemson South Carolina 29634 USA; ^2^ Advanced Plant Technology Clemson University Clemson South Carolina 29634 USA; ^3^ Center for Plant Science Innovation and Department of Agronomy and Horticulture University of Nebraska‐Lincoln Lincoln Nebraska 68588 USA; ^4^ Carolina Seed Systems Darlington South Carolina 29532 USA; ^5^ Pee Dee Research and Education Center Clemson University Florence South Carolina 29506 USA; ^6^ Feed the Future Innovation Lab for Crop Improvement Cornell University Ithaca New York 14850 USA

**Keywords:** Sorghum bicolor, whole‐genome sequencing, diversity panel, copy‐number variants, genomic prediction, genome‐wide association studies, selection signatures, pleiotropy, genotyping‐by‐sequencing, pan‐genome

## Abstract

Association mapping panels represent foundational resources for understanding the genetic basis of phenotypic diversity and serve to advance plant breeding by exploring genetic variation across diverse accessions. We report the whole‐genome sequencing (WGS) of 400 sorghum (*Sorghum bicolor* (L.) Moench) accessions from the Sorghum Association Panel (SAP) at an average coverage of 38× (25–72×), enabling the development of a high‐density genomic marker set of 43 983 694 variants including single‐nucleotide polymorphisms (approximately 38 million), insertions/deletions (indels) (approximately 5 million), and copy number variants (CNVs) (approximately 170 000). We observe slightly more deletions among indels and a much higher prevalence of deletions among CNVs compared to insertions. This new marker set enabled the identification of several novel putative genomic associations for plant height and tannin content, which were not identified when using previous lower‐density marker sets. WGS identified and scored variants in 5‐kb bins where available genotyping‐by‐sequencing (GBS) data captured no variants, with half of all bins in the genome falling into this category. The predictive ability of genomic best unbiased linear predictor (GBLUP) models was increased by an average of 30% by using WGS markers rather than GBS markers. We identified 18 selection peaks across subpopulations that formed due to evolutionary divergence during domestication, and we found six F_st_ peaks resulting from comparisons between converted lines and breeding lines within the SAP that were distinct from the peaks associated with historic selection. This population has served and continues to serve as a significant public resource for sorghum research and demonstrates the value of improving upon existing genomic resources.

## Significance Statement

The whole‐genome sequencing of the sorghum association panel, which represents the most heavily utilized panel in sorghum breeding, will serve as a pivotal resource in the sorghum community to provide improved genotype‐to‐phenotype associations, genomic prediction, and pan‐genome graph construction as demonstrated here. These data will support ongoing and future research focused on gene discovery and trait introgression in the sorghum community and further provide insights into the historic selection and domestication of this multi‐purpose crop.

## INTRODUCTION

Sources of natural genetic variation are foundational to crop improvement as they can be used in the genetic dissection of pivotal traits and the development of breeding populations. Along with advances in modern statistics and technology, maintenance and expansion of genetic diversity within available germplasm is paramount to the advancement of crop improvement. Since the early development of association mapping populations in maize (*Zea mays* L.) (Thornsberry et al., 2001), these panels have served to propel plant breeding by exploring genetic variation across diverse accessions with distinct history of evolutionary divergence and local adaptation. Now, two decades after that initial maize panel, all major cereal grains have association panels, including barley (*Hordeum vulgare* L.) (Stracke et al., 2009), maize (Romay et al., 2013; Thornsberry et al., 2001; Yang et al., 2011), rice (*Oryza sativa* L.) (Zhao et al., 2011), sorghum (*Sorghum bicolor* (L.) Moench) (Brenton et al., 2016; Casa et al., 2008; Morris et al., 2013), and wheat (*Triticum aestivum* L.) (Garcia et al., 2019; Lopes et al., 2012). Association mapping panels leverage the existing natural variation – both genetic and phenotypic – of a population to resolve complex trait variation to the genomic features influencing the phenotypic variance. As such, the diversity of germplasm in an association panel is vital to increase our understanding of causal biological mechanisms and translate to crop improvement (Gupta et al., 2014).

The Sorghum Association Panel (SAP), the first sorghum diversity panel, is composed of temperate‐adapted breeding lines, as well as converted (photoperiod‐insensitive) tropical accessions from the Sorghum Conversion Program (SCP) (Klein et al., 2008; Stephens et al., 1967). The accessions in the SAP were selected to maximize the genetic and phenotypic diversity of the panel as well as capture accessions that are important for understanding the demographic history and historical breeding importance based on known resistances or tolerances to abiotic and biotic stresses (Casa et al., 2008). Sorghum's broad geographic distribution (Casa et al., 2008; De Wet & Harlan, 1971) and carbon‐partitioning regimes (Boatwright et al., 2021) have resulted in two classification systems that distinguish accessions based on variation by race and carbon partitioning, with race representing the predominant classification system in the SAP. Sorghum is classified into five botanical races: bicolor, caudatum, durra, guinea, and kafir, which are thought to have resulted through multiple domestication and adaptation events across different clines (Harlan & Stemler, 1976; Morris et al., 2013).

The SAP was originally genotyped using simple sequence repeat markers (Casa et al., 2008) and later sequenced using restriction site‐based genotyping‐by‐sequencing (GBS) to obtain low‐coverage single‐nucleotide polymorphism (SNP) data (Boyles et al., 2016; Morris et al., 2013). However, as sequencing costs have continued to decline, large association panels such as the SAP can be sequenced using whole‐genome sequencing (WGS) at a lower cost to generate reliable genomic variants at high density for applications in genetics and breeding (Causse et al., 2013). These high‐throughput sequencing data can enable computational analyses for genomics‐assisted breeding with the help of diverse variant types including SNPs, insertions/deletions (indels), and larger structural variants (SVs). These variant types are also valuable for understanding genetic diversity when combined into variant graphs, also known as pan‐genomes (Hickey et al., 2020; Jensen et al., 2020). Increased variant density also permits the identification of causal variants as opposed to variants that simply lie in linkage disequilibrium (LD) with causal variants. The application of WGS to the SAP will increase the power and utility of the SAP, just as GBS improved upon SSR markers, and serve to expand upon the identified genetic diversity that facilitates genome‐wide association mapping (GWAS) and genomic selection in sorghum (Ersoz & Yu, 2009).

A variant graph better represents the true diversity of variant information across a population and ameliorates issues associated with mapping bias inherent in traditional reference‐based genomics (Bayer et al., 2020; Jensen et al., 2020). When a traditional reference genome is used, variants missing from the reference, such as those arising from recent duplications or deletions, cannot be identified by quantitative trait locus (QTL) mapping or GWAS (Della Coletta et al., 2021). Such limitations are particularly pervasive when studying diverse accessions, and while using a different reference genome can circumvent this issue, variant graphs are particularly well suited to capture this information and significantly reduce mapping biases (Della Coletta et al., 2021). Additionally, tools such as the GATK (McKenna et al., 2010; Poplin et al., 2017) permit joint calling of variants across samples in a population to increase the power to detect true variants, and when indels are present, the joint calling methods can assess variants through localized assembly from the read data to reduce the impact of read mapping biases on variant discovery (Poplin et al., 2017). Together, these tools permit robust variant discovery along with development of a diverse pan‐genomic reference for future studies in sorghum.

In this study, we report the development and use of high‐density genomic variants including SNPs, indels, and copy number variants (CNVs) for population and translational genomic analysis using WGS. The application of high‐throughput genotyping and robust variant discovery for the highly diverse SAP provides the genomic resources necessary for acceleration of gene discovery, genomics‐assisted breeding, and genetic engineering toward improved cultivar development and carbon‐negative agriculture. We demonstrate the value of the WGS genomic resource over GBS markers through comparative advantages in the identification of novel genomic associations and increased accuracy in genomic prediction for various traits.

## MATERIALS AND METHODS

### Plant material and datasets

A total of 400 accessions in the United States SAP (Casa et al., 2008) were obtained through the Agricultural Research Service Germplasm Resources Information Network (ARS‐GRIN) (http://www.ars‐grin.gov) (File S1). Seedlings were grown by sowing three to five seeds from each accession in a plastic pot in the Biosystems Research Complex greenhouse at Clemson University, Clemson, SC. Tissue was collected from 2‐week‐old seedlings and lyophilized for three days in a LABCONCO FreeZone 4.5‐L −50°C benchtop freeze dryer prior to DNA extraction. Phenotypic data for all the traits used in genome‐wide association and prediction analyses were derived from previously published datasets (Boyles et al., 2016; Boyles et al., 2017; Sapkota, Boatwright, et al., 2020; Sapkota, Boyles, et al., 2020). We accessed the publicly available GBS data for the SAP to conduct comparative analyses between our WGS data and GBS marker data (Boyles et al., 2016; Morris et al., 2013).

### Whole‐genome sequencing data production and processing

WGS data was generated by RAPiD Genomics, Gainesville, FL using DNA extracted from lyophilized leaf tissue. WGS libraries were paired‐end sequenced at approximately 30× coverage using an Illumina NovaSeq sequencer resulting in 2 × 150‐bp reads. WGS reads were cleaned using fastp (Chen et al., 2018) before aligning with BWA version 0.7.17 (Li & Durbin, 2010) to the BTx623 version 3.1 annotated reference genome (McCormick et al., 2018) obtained from Phytozome (https://phytozome‐next.jgi.doe.gov/) (Goodstein et al., 2012). Both SNP and indel variants were called using the Genome Analysis Toolkit (GATK) pipeline version 4.1.7.0 (McKenna et al., 2010) following GATK best practices (DePristo et al., 2011; Van der Auwera et al., 2013). Joint calling in the GATK was used to increase sensitivity for low‐frequency variants, to better distinguish between homozygous reference sites and sites with missing data, and to maximize SNP fidelity by allowing accurate error modeling (Poplin et al., 2017). Variants were subsequently quality filtered using QD < 2.0, InbreedingCoeff < 0.0, QUAL < 30.0, SOR > 3.0, FS > 60.0, MQ < 40.0, MQRankSum < −12.5, and ReadPosRankSum < −8.0. Beagle version 5.1 was used to impute missing genotype data for biallelic SNPs in the variant call format (VCF) file resulting from the GATK pipeline. SNP density plots were generated using R‐CMplot version 3.6.0 (https://github.com/YinLiLin/R‐CMplot) in the R programming language (Core, 2019).

The inbreeding coefficient and nucleotide diversity were calculated using VCFtools version 0.1.16 (Danecek et al., 2011). Nucleotide diversity was estimated using a non‐overlapping 1‐Mb sliding window and plotted using Circos (Krzywinski et al., 2009). The effects of SNPs and indels were predicted using snpEff (Cingolani et al., 2012) and general variant statistics were collected using BCFtools (Danecek et al., 2021). The variant metrics and predictions were collected and plotted using MultiQC (Ewels et al., 2016). GBS and WGS variant effect counts were collected from the snpEff results and plotted using Excel. To compare GBS and WGS coverage, SNPs were counted with bin sizes of 5, 10, 15, 20, and 40 kb using a custom Python script and plotted using *ggplot2* (Wickham, 2016). The LD decay plot was generated using PopLDdecay v3.40 (Zhang et al., 2019) and custom R scripts (Core, 2019). HaploBlocker v1.6.06 (Pook et al., 2019) was used to identify subgroup‐specific haplotype blocks where a haploblock is defined as a sequence of genetic markers that occurs at least five times within the population. Each accession is then checked to determine if they contain a similar sequence of markers, which serves to screen the population in a group‐wise, identity‐by‐descent manner (Pook et al., 2019). The number of ancestral populations represented by the SAP was estimated using the R package *adegenet* v2.1.3 (Jombart, 2008) where a discriminant analysis of principal components (DAPC) was performed for 1–12 clusters and the Bayesian information criterion (BIC) was used to identify the optimal number of clusters to describe the population. Subsequently, ADMIXTURE v1.3.0 was executed using the number of clusters estimated from DAPC as K to visualize the degree of admixture across the SAP (Alexander et al., 2009; Alexander & Lange, 2011). Gene network analysis and enrichment were performed using the STRING database (https://string‐db.org/) (Franceschini et al., 2013). STRING represents a database of known and predicted protein–protein interactions and provides network models and enrichment statistics. Functional enrichment of gene ontologies was filtered using a false discovery rate of 0.01, and the minimum required interaction score was set to high confidence (0.7).

Numerous tools can detect CNVs from WGS data, but the complexity of plant data can complicate accurate variant calling, especially when many tools were designed with default settings suited for human data (Wijfjes et al., 2019). As such, we called CNVs using Hecaton v0.3.0 (Wijfjes et al., 2019), which uses multiple CNV tools, including DELLY v0.8.5 (Rausch et al., 2012), GRIDSS v2.0.1 (Cameron et al., 2017), LUMPY v0.2.13 (Layer et al., 2014), and Manta v1.4.0 (Chen et al., 2016), to detect CNVs before using a random forest model to distinguish probable false positive from true positive variant calls based on a pre‐trained model specific to plants. Hecaton has been shown to outperform current methods when applied to short‐read WGS data of Arabidopsis, maize, rice, and tomato (*Solanum lycopersicum* L.) (Wijfjes et al., 2019).

### Genome‐wide analysis for selection signatures

Subpopulations identified using ADMIXTURE analysis (K = 6) were used to estimate F_st_ according to the methods of (Weir & Cockerham, 1984) using the vcftools function ‐‐*weir*‐*fst*‐*pop* on the SNP variants after filtering for minor allele frequency (MAF) > 5% for each subpopulation (Danecek et al., 2011). A window size of 1 Mb with a step size of 100 kb was used for calculation. F_st_ estimates were calculated for each subpopulation against all other subpopulations, and the mean F_st_ for a subpopulation at a genomic window was computed as averaged F_st_ of a subpopulation against all other subpopulations for that genomic window. Additionally, we also computed F_st_ between accessions derived from sorghum conversion program (*N* = 240) and temperate breeding lines (*N* = 96) within the panel using the same parameters mentioned above (File S1). Tajima's D for the whole panel was calculated for 1‐Mb non‐overlapping windows using the vcftools function ‐‐*TajD*.

### Genome‐wide association studies

The software GEMMA v0.98.1 (Zhou et al., 2013; Zhou & Stephens, 2012) was used for GWAS. The imputed VCF file containing biallelic SNPs was converted to PLINK format using PLINK (Purcell et al., 2007), and GEMMA was then used to calculate a standardized relatedness matrix for linear mixed modeling on the filtered data (−‐*miss* = 0.3 ‐‐*maf* = 0.05). All models were run using a MAF filter of 0.05 and linear mixed models (LMMs) of the following the form:

(1) $$y=X\beta +Zu+\varepsilon ;u∼N\left(0,G\right);\varepsilon ∼N\left(0,R\right)$$

where *y* is a vector of phenotypic values for a single trait, X is a numeric genotype matrix generated from the SNPs, β represents an unknown vector of fixed effects and includes the effect size for each SNPs, Z is the design matrix for random effects, *u* is an unknown vector of random effects, and ε is the unknown vector of residuals. These models test the alternative hypothesis H_1_: β ≠ 0 against the null hypothesis H_0_: β = 0 for each SNP. Manhattan and Q‐Q plots were generated using R‐CMplot version 3.6.0 and ggplot2 version 3.3.5. GEMMA was also used to run Bayesian sparse LMMs (BSLMMs) to better identify causative variants, with a probit model used for binary phenotypic data. The BSLMM model assumes fixed effects are distributed according to the sparse prior β ∼ πN(0, σ^2^_a_τ^−1^) (Zhou et al., 2013). LD statistics were calculated for significant loci using PLINK v1.9 (Purcell et al., 2007).

A multivariate adaptive shrinkage approach was used to assess the degree of pleiotropic effects across the traits. Using the estimated effect sizes and standard errors for each marker from the GEMMA LMMs, a local false sign rate (lfsr) was calculated on a condition‐by‐condition basis using ashr in R (Stephens, 2016) to filter variants based on lfsr < 0.1. The lfsr represents the probability of incorrectly assigning the sign of an effect. The lfsr has been demonstrated to serve as a superior measure of significance over traditional multiple‐testing corrections such as Bonferroni or false discovery rate (Benjamini & Hochberg, 1995) due to its general applicability and robust estimation process (Stephens, 2016). A control set of 1 200 000 random markers was also generated from the full set of markers to estimate the covariance between markers for each phenotype. From this control set, a correlation matrix was estimated using *MashR* (Urbut et al., 2019) to control any confounding effects arising from correlated variation among the traits. Using both canonical and data‐driven covariance matrices, we tested for pleiotropy across traits. Posterior probabilities were estimated for each marker using a mash model with all marker tests. The *CDBNgenomics* R package (MacQueen et al., 2020) was then used to extract Bayes factors and generate a Manhattan plot of the mash results where Bayes factors > 3 were considered significant for pleiotropic effects.

### Genome‐wide prediction

Previously published SAP phenotypic data (Boyles et al., 2016; Boyles et al., 2017; Sapkota, Boatwright, et al., 2020; Sapkota, Boyles, et al., 2020) were used to compare genomic prediction results between WGS and GBS marker data using the R package *sommer* (Covarrubias‐Pazaran, 2016). A genomic best linear unbiased prediction (GBLUP) model of the following form was fit:

(2) $$y_{i}=\mu +g_{j}+\epsilon_{\mathrm{ij}}$$

where *y*_*i*_ is a vector of BLUPs for trait *i*, μ is the overall mean, g_*j*_ is a vector of random effect of genotypes with g ∼ N(0, Aσ^2^_g_), where σ^2^_g_ is additive genetic variance and A is the realized additive relationship matrix calculated from an *n* × *m* genotype matrix with *n* genotypes and *m* markers using the *A.mat* function from the *rrBLUP* package (Endelman & Jannink, 2012), and ɛ_*ij*_ is a vector of residuals that are identical and independently distributed with ɛ ∼ N(0, Iσ^2^_e_), where σ^2^_e_ is the residual variance and I is an identity matrix.

Model performance was assessed using a 10‐fold cross‐validation where nine datasets were used for model training and the remaining dataset was used as the testing set. Predictive ability was calculated as Pearson's correlation coefficient between predicted and observed values for the testing set. A total of 100 iterations were run for each trait using the *set.seed* function in R for sampling seeds from 123 to 222. The predictive ability was compared between GBS and WGS using the pairwise *t*‐test (*pairwise.t.test* function in R), and *P*‐values were adjusted using Benjamini–Hochberg correction (Benjamini & Hochberg, 1995).

### Variant graph construction

A variant graph was constructed using *vg* (Hickey et al., 2020), which incorporated the Sorghum BTx623 reference genome together with SAP variants (SNPs, indels, and CNVs) called using the GATK (McKenna et al., 2010) and Hecaton (Wijfjes et al., 2019) pipelines. Individual chromosomes were constructed using *vg construct* with the options ‐‐*handle*‐*sv* and ‐‐*node*‐*max* of 32. After chromosome‐level construction, each subgraph was given unique node identifiers using *vg ids* before building a single joint variant graph in XG format, which permitted querying of the variant graph and alignment of read data. Reads obtained from (Mace et al., 2013) were then aligned using the joint variant graph and *vg map* with default parameters. The resulting GAM file was quality filtered and used to calculate read support with *vg pack* before calling variants on individual samples with *vg call*.

## RESULTS

### SNPs, indels, and CNVs

**Figure 1:**
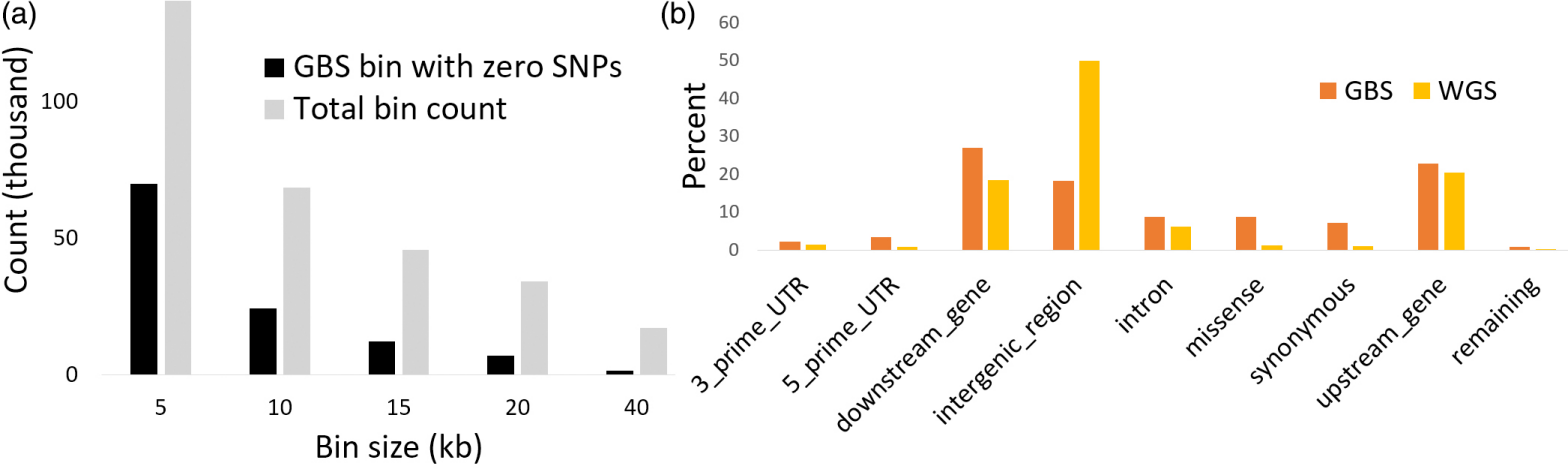
Comparison of genotype‐by‐sequencing (GBS) and whole‐genome sequencing (WGS) single‐nucleotide polymorphism (SNP) distributions. Total bin count and counts for bins where GBS data lacked a SNP but WGS had SNPs across different bin sizes are shown in panel (a), and panel (b) includes the percentage of variants across the major genic and intergenic regions for both GBS and WGS data. [Colour figure can be viewed at wileyonlinelibrary.com]

**Figure 2:**
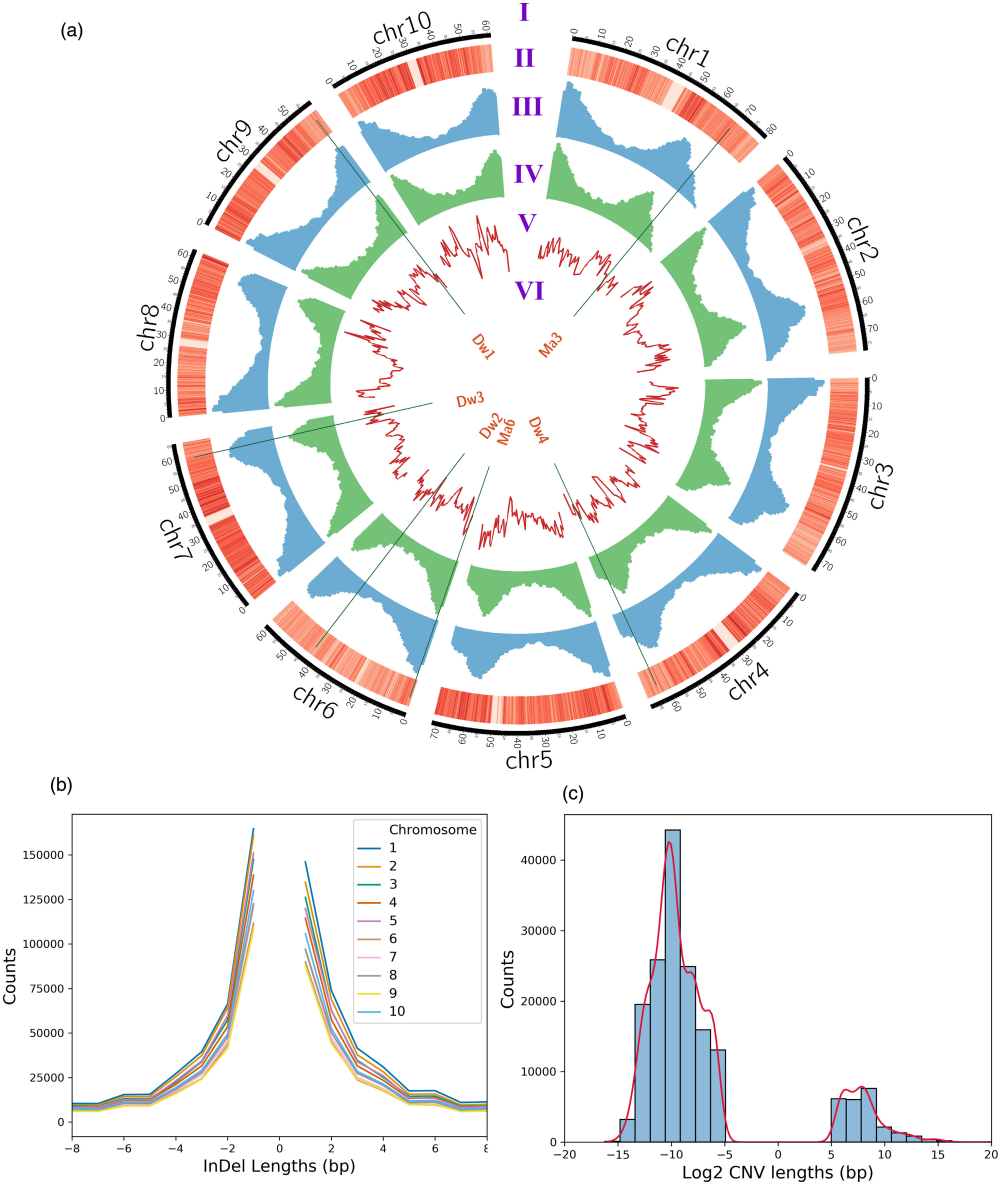
Genome‐wide variant coverage and diversity. (a) Circos plot containing tracks for the sorghum karyotype (a), SNP density heatmap (b), indel histogram (c), copy number variant (CNV) histogram (d), nucleotide diversity (e), and genes (f). (b) Line plot demonstrating the total number of indels across varying indel lengths. (c) Histogram and kernel density estimate for CNV counts across varying log2 CNV lengths. [Colour figure can be viewed at wileyonlinelibrary.com]

We sequenced 400 accessions from the SAP at an average coverage of 38×, ranging from 25× to 72× (Figures S1 and S2), representing a total of approximately 82 billion reads or approximately 11 trillion bases after quality control. Reads exhibited highly consistent GC content across all samples (Figure S3). The SAP genotypic data contained 43 983 694 variants, which includes SNPs (approximately 38 million) and indels (approximately 5 million) identified using the GATK pipeline and CNVs (approximately 170 000) called using Hecaton (Table S1). A total of 19 708 560 SNPs passed quality filtering based on variant likelihood metrics and 5 420 745 SNPs of these SNPs exhibited MAF > 5%. Using the quality filtered SNPs, snpEff estimated the overall transition/transversion ratio at 1.89 (Figure S4). Approximately 50% of the predicted variant effects fell into intergenic regions, with 20% occurring in upstream regions, 19% in downstream regions, and 11% in the remaining genic regions. The variant effects were distributed at a low frequency among low (451 663), moderate (439 159), and high impacts (15 019). In total, 9978 genes overlapped with high‐impact variants while low‐ and moderate‐impact variants accounted for nearly all annotated genes at 44 219 and 44 752 genes, respectively. To compare the coverage quality of our WGS data to existing GBS marker data, we binned variants in 5‐kb windows across the genome. In our comparison, we observed that the GBS data did not have any variants for half of the bins across the sorghum genome, whereas WGS had at least one variant in those bins (Figure 1a). Consistent with the methylation‐sensitive nature of the *Ape*KI enzyme used to generate the GBS marker data, the GBS markers exhibited a strong bias toward genic regions while the distribution of variants in WGS data largely mirrored the overall proportion of genic and intergenic sequences in the sorghum genome with approximately 50% of total markers located in the intergenic regions, which were defined as variants between annotated gene models or between a gene and the end of a chromosome (Figure 1b). In general, the WGS data showed higher variant density on chromosome arms and in telomeric regions than in pericentromeric and centromeric regions (Figure 2a).

The genome‐wide average nucleotide diversity was 2.4 × 10^−3^ for the entire population with variation in SNP density across the genome showing telomeric regions accumulate more mutations than centromeric regions because of higher recombination rates (Figure 2a). A total of 2 652 314 indels were identified after quality filtering with a strong telomeric bias in distribution (Figure 2a). The majority (82.56%) of indels were less than 15 bp in length (Figure 2b; Figure S5), but the largest indel was 387 bp. Larger CNVs identified using Hecaton, ranging from 49 bp to 1 Mb, were retained for analyses, whereas all CNVs over 1 Mb in length were deemed false positives due to the limitations of short‐read data (Wijfjes et al., 2019). We identified nearly seven times more CNVs compared to a previous study in sorghum (Songsomboon et al., 2021) likely due to the variant callers used as Songsomboon et al. (Songsomboon et al., 2021) only used LUMPY, but we used LUMPY and three other variant callers within Hecaton. The identification of indels is relative to the reference genome used. So, while it is possible that the BTx623 reference may have abnormally high copy numbers, the likely explanation for such high prevalence of deletions over insertions is that the variant callers can more easily identify deletions due to the loss of coverage associated with deletions. Not only did the distribution of CNV types show a preponderance of deletions over insertions (Figure 2c), these variant types were also more densely distributed around telomeric regions (Figure 2a), which is consistent with previous observations in sorghum (Songsomboon et al., 2021).

### Population structure, haplotype blocks, and variant graph construction

We estimated the LD decay distance for individual chromosomes as well as for the whole genome because LD influences the genetic mapping resolution and is essential in haplotype construction. The genome‐wide average distance at which LD decayed to *r* ^2^ < 0.2 was approximately around 20 kb and the LD decay leveled out around 150 kb (Figure S6). Chr6 exhibited consistently higher LD compared to the other chromosomes, which is consistent with previous reports of limited recombination in Chr6 (Hu et al., 2019; Wang et al., 2013), and as a result, the average physical size of estimated haplotype blocks was larger for Chr6.

**Figure 3:**
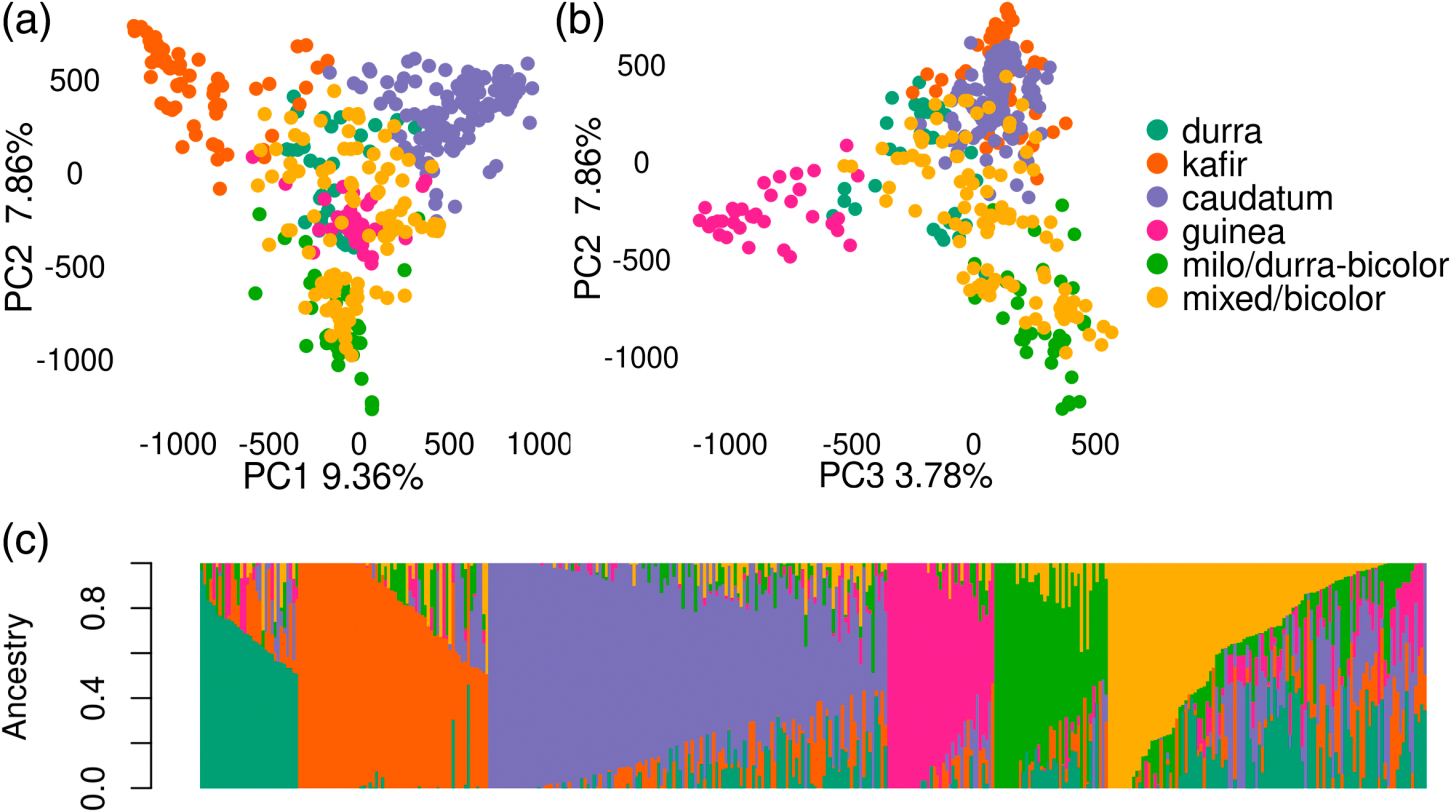
Population structure within the sorghum association panel using principal component analysis and an admixture model (*K* = 6). Subpopulations were labeled with corresponding botanical races or sorghum types that predominated for a given subpopulation. Subplots represent the (a) projection of panel accessions by the first principal component (PC1) and PC2, (b) projection by PC2 and PC3, and (c) the degree of admixture across the subpopulations using consistent subpopulation colors across all subplots. [Colour figure can be viewed at wileyonlinelibrary.com]

DAPC estimated the optimal number of clusters to be eight, but there was no significant difference in BIC for cluster counts between 6 and 11 (Figure S7, Figure S8). For simplicity, we opted to use the lowest number of population clusters (K = 6) with lower BIC for subsequent ADMIXTURE analysis. The subpopulation grouping in the population structure analysis led to four clusters that correspond to the four botanical races of sorghum (caudatum, kafir, guinea, and durra) (Figure S9). The fifth subpopulation cluster consisted of several durra‐bicolor accessions that were historically categorized as milo and therefore we referred to that subpopulation/racial type as milo (Figure S9). The sixth subpopulation consisted of some durra accessions but were mostly composed of mixed‐race accessions and the accessions classified as bicolor, which is thought to be the early sorghum domesticate and therefore does not form a separate subpopulation cluster (Harlan & Stemler, 1976; Sapkota, Boyles, et al., 2020). The first 10 components in principal component (PC) analysis accounted for about 36% of the genomic variation, with the first three PCs explaining 9.36%, 7.86%, and 3.78% of the variation, respectively. The first PC separated kafir accessions from caudatum, PC2 separated kafir, caudatum, and durra from milo, and PC3 distinguished guinea accessions from all other accessions (Figure 3a,b).

Ancestral population admixture was consistent with observed historical patterns among the sorghum races with greater admixture among approximately one fourth of the accessions (Figure 3c). We also estimated haplotype blocks to understand the patterns of linkage across the sorghum genome and the potential effects linkage may have on selection. A total of 35 029 haplotype blocks with an average length of 40 kb were identified using Haploblocker (Pook et al., 2019), which examines linkage across population subgroups to estimate haplotype blocks. Over 4000 blocks were identified in chromosomes 2, 4, and 5, but fewer than 3000 blocks were identified in chromosomes 6, 7, and 9 (Table S2). The remaining chromosomes contained between 3000 and 4000 haplotype blocks based on the SAP accessions. These results indicate that selection has likely been stronger on chromosomes 6, 7, and 9 and resulted in fewer and larger haplotype blocks, which is consistent with the selection of the major dwarfing loci on these chromosomes as part of the sorghum conversion program.

We used the variant graph software *vg* to successfully generate a pan‐genome, examine the locus haplotype structure in the SAP, map WGS reads from Mace et al. (Mace et al., 2013), and call SNPs, indels, and CNVs from the sample alignments using the external read data. The mapping of external data serves to demonstrate the direct applicability of these SAP data to support pan‐genomics and variant calling from a pan‐genome. We obtained approximately 3 million variants per sample that subsequently reduced to approximately 1 million per sample following quality filtering. As the sequencing data from Mace et al. represent similar coverage, differences in variant coverage likely arise from differences in methods used in variant calling. Compared to joint calling via the GATK pipeline and CNV calling using Hecaton, *vg* was able to call all variant types in a single run with a significantly shorter runtime than the alternative approaches employed above. However, the total number of variants obtained was an order of magnitude lower. This is likely due to several factors including increased power to detect variants when sharing haplotype and coverage information in joint calling (Poplin et al., 2017), the use of multiple CNV callers by Hecaton (Wijfjes et al., 2019), and the state of development for the variant calling methodology in *vg* (Hickey et al., 2020).

### Genomic signatures of selection

The development of sorghum racial types is thought to be an outcome of multiple domestication events and subsequent local adaptations leading to the distinct population structure observed (Harlan & Stemler, 1976; Kimber et al., 2013; Morris et al., 2013). The resulting population structure is congruent with these racial types, as evidenced by the population (SAP) we have sequenced in this study. We attempted to identify and contrast signatures of historic selections during domestication to those that have occurred recently due to the photoperiod conversion of tropical accessions and/or selections made by breeding programs. We computed genome‐wide F_st_ between racial subgroups arising from evolutionary diversification to identify signatures of historic selection, whereas genome‐wide F_st_ peaks between the latter groups (converted and bred) were used to identify signatures of artificial selection due to temperate conversion and/or breeding.

Several regions across the genome showed selective sweeps (F_st_ peaks) for subpopulations identified using population structure analysis (Figure S10). There were 18 genomic regions with strong selection peaks, of which four regions across three chromosomes (Chr2, Chr3, and Chr8) had common F_st_ peaks in at least three subpopulations (Table 1, Figure S10). The selected region around 45–54 Mb of Chr2 that had strong peaks for all subpopulations but the kafir subpopulation has around 279 genes and seven QTL previously mapped to this region (Table 1). Among the seven QTL in this region were three mapped for tannin by Rhodes et al. (Rhodes et al., 2014) and one each for amylose (Chen et al., 2019), panicle length (Morris et al., 2013), seedling survival (Bekele et al., 2014), and anthracnose resistance (Cuevas & Prom, 2020). Among the genes within this region, 24 genes had coiled‐coil domains and showed significant enrichment based on the gene network analysis site STRING (https://string‐db.org/). Another commonly selected region around 21–29 Mb of Chr3 had 68 genes that included several genes involved in biological regulation and molecular function including photosynthesis, but no QTL was previously mapped in the region (Table 1).

**Table 1:** Regions with strong (mean + 3 × standard deviation) selection sweeps based on F_st_ estimates for racial subpopulations identified from admixture analysis. Start and end positions are measured in Mb

Race/subpopulation	Chr^a^	Start	End	Genes^b^	QTL^c^
Durra	1	20.0	22.2	195	16
Mixed	1	40.8	41.8	0	0
Milo	1	70.3	71.3	193	43
Caudatum, durra, guinea, milo, mixed	2	45.2	53.6	279	7
Guinea	2	70.9	72.7	335	50
Milo	2	76.9	78.7	159	0
Caudatum, durra, milo, mixed	3	21.2	29.4	68	0
Durra	3	14.1	15.2	74	26
Guinea	3	17.1	19.6	93	0
Milo, guinea, caudatum	3	40.2	45.7	88	0
Durra	3	48.5	50.5	69	35
Caudatum	4	64.4	66.0	255	71
Milo	5	19.0	20.6	58	0
Kafir	5	24.2	55.0	272	16
Caudatum, durra, milo	8	7.8	11.4	121	14
Durra	8	18.0	23.0	38	0
Guinea	10	39.7	40.8	4	0
Milo	10	61.1	62.2	19	2

^a^ Chromosome abbreviated as Chr.

^b^ Number of genes based on sorghum BTx623 V3.1.1 annotation (phytozome.org).

^c^ QTL, quantitative trait loci; number of QTL within the genomic region based on the Sorghum QTL atlas (https://aussorgm.org.au/sorghum‐qtl‐atlas/).

In general, the milo subpopulation had the highest number (eight) of significant sweeps, followed by durra (seven), while kafir had the lowest (1) (Table 1). One potential explanation for this could be the evolutionary and demographic history of these racial types. The kafir race is the youngest among the botanical sorghum races while the durra race, which includes the milo type, is believed to have independently formed away from the African content (Kimber et al., 2013). The only strong selection sweep identified in kafir was unique to the subpopulation and ranged from 24 to 55 Mb of Chr5 (Table 1). The caudatum subpopulation had a selection peak at 64–66 Mb of Chr4 near the *Tan1b* locus (Sobic.004G280800), which is associated with tannin/polyphenolic content (Wu et al., 2012). Milo showed strong selection between 70 and 71 Mb of Chr1, which is near the *Y* locus (Sobic.001G397900) that  encodes for yellow pericarp (seed color) in sorghum (Ibraheem et al., 2010) and was captured using testa pigmentation (Figure S10). Durra, the subpopulation that is closely related to most of the accessions within the milo subpopulation, also had a minor peak around this region (Figure S10). The only unique selection peak for the mixed subpopulation occurred around the non‐genic region ranging from 41 to 42 Mb of Chr1. In general, most of these genomic regions with strong selection signatures had many characterized genes and several previously mapped QTL in and around these regions (Table 1).

**Figure 4:**
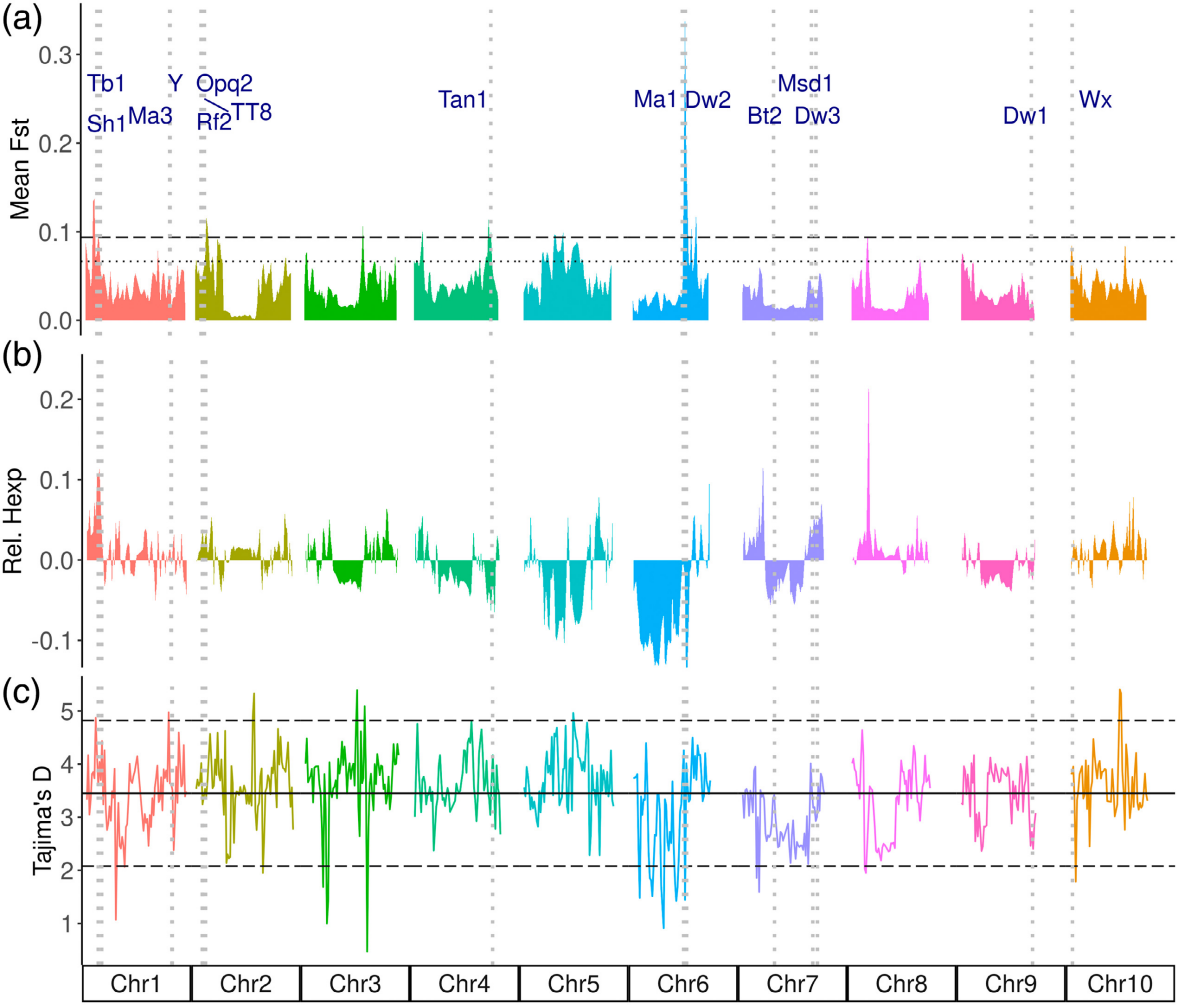
Genome‐wide signatures of selection. (a) The mean F_st_ across the sorghum genome between tropical converted and temperate breeding acessions. (b) Expected heterozygosity in the converted group relative to the bred group. (c) Genome‐wide Tajima's D estimates. The horizontal lines show mean and standard deviations of the estimates; solid black lines show the genome‐wide average Tajima's D; dotted lines show the mean plus 1 standard deviation; and long dashed lines show the mean plus 2 standard deviations of the estimates. Vertical dotted lines show genes and loci related to height, maturity, and other domestication related traits. [Colour figure can be viewed at wileyonlinelibrary.com]

Based on the F_st_ estimates, a total of six genomic regions showed strong selection sweeps between the accessions in the converted and bred groups (Figure 4a). The strongest peak was observed around 41–47 Mb of Chr6. This region contains major‐effect maturity (*Ma1*: Sobic.006G057866) and height (*Dw2*: Sobic.006G067700) genes that were introgressed for early maturity and short stature, respectively, during sorghum conversion (Figure 4a). Another region that showed a strong selection sweep was the region around the *Tan1* gene, which is associated with tannin content (Figure 4a). Additional peaks were observed in the beginning of Chr1, Chr2, Chr4, and Chr8. The genomic region around the waxy locus (Sobic.010G022600) also showed a minor peak that was one standard deviation above the mean but did not reach two standard deviations (Figure 4a). We calculated expected heterozygosity (2pq) for the converted and bred groups on a per‐site basis using allele frequencies (p and q) of SNPs at a given site. Figure 4b shows expected heterozygosity across each site for accessions in the converted group relative to the accessions in the bred category. The variation in relative heterozygosity for the two groups was consistent with the distribution of F_st_ peaks between the two groups (Figure 4a,b).

The genomic region around the dwarfing and maturity genes showed stronger bottlenecks for nucleotide diversity and Tajima's D for the SAP (Figures 1a and 4c), and the whole‐genome average value for Tajima's D was 3.45, indicating that there are fewer rare alleles across the genome because of extensive inbreeding across the population. Most of the genomic regions showed Tajima's D above the mean value indicating balancing selection while some regions particularly at Chr1, Chr3, and Chr6 showed strong bottlenecks indicative of purifying selection (Figure 4c). The regions in the middle of Chr6 and Chr7 showed a strong bottleneck in Tajima's D and expected heterozygosity for converted lines compared to the breeding lines (Figure 4b, Figure S11).

### Genome‐wide association for plant height and tannin content

**Figure 5:**
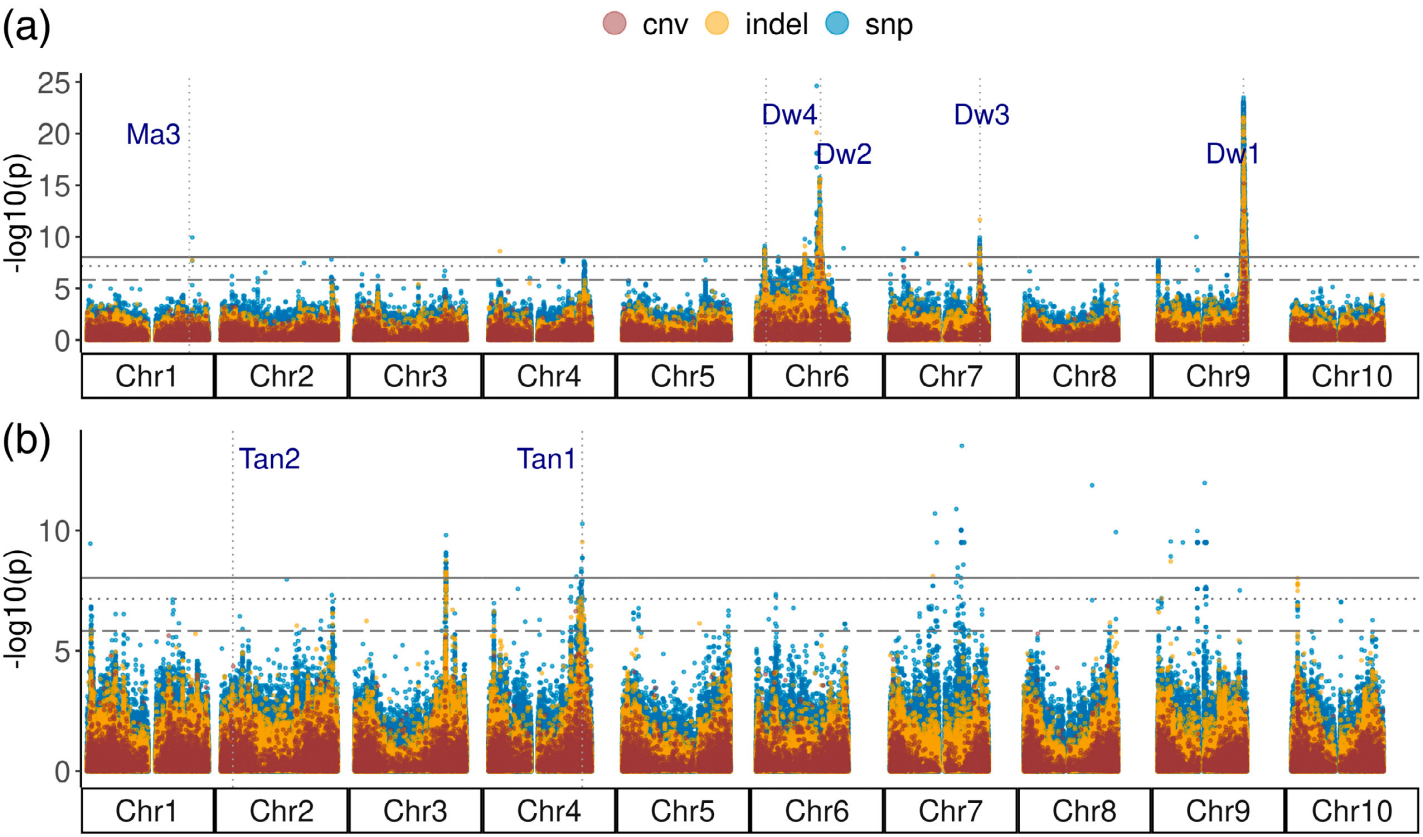
Genome‐wide associations for plant height (a) and tannin content (b) using linear mixed models in GEMMA. Horizontal lines with solid, dotted, and dashed patterns represent the Bonferroni‐adjusted threshold of 0.05 for SNPs, indels, and CNVs, respectively. Vertical dotted lines indicate the positions of known genes and loci for height (Dw), maturity (Ma), and tannin (Tan). [Colour figure can be viewed at wileyonlinelibrary.com]

In sorghum, plant height (PH) and tannin content have been thoroughly examined due to their significant impacts on both historic selection (Wu et al., 2019) and modern agriculture (Dillon et al., 2007). PH in sorghum is genetically controlled by multiple genes including three predominant loci (*Dw1*: Sobic.009G229800, *Dw2*: Sobic.006G067700, and *Dw3*: Sobic.007G163800) explaining a large majority of the phenotypic variation in our population. All three major dwarfing loci showed significant association for PH using all three variant types independently (Figure 5a). For both SNP and indel variants, we also identified significant association for the previously reported *Dw4* locus, which occurs at approximately 6.6 Mb on Chr6 (Miao et al., 2020; Morris et al., 2013). Additionally, we identified a significantly associated indel at approximately 6.5 Mb on Chr4 that overlaps with a previously identified QTL for total PH, flag leaf height, and flag leaf to apex interval (Figure 5a, Table 2) (Li et al., 2015). This locus contains eight genes, two of which are functionally annotated (Table 2). Among them, one gene is associated with the plant viral response, and the other gene encodes an F‐box protein, which is known to regulate plant vegetative and reproductive growth. A novel locus that is approximately 2 Mb downstream of maturity gene *Ma3* (Sobic.001G394400) also showed a significant association with PH for SNP as well as indel variants (Figure 5a, Table 2). This locus is a hotspot for heat shock protein 70 (HSP70) with five HSP70 proteins within 20 kb and more than 10 HSP70 proteins within 100 kb of the associated SNP peak.

**Table 2:** Novel putative associated loci identified using whole‐genome sequencing (WGS) data. Peak coordinates are represented in bases

Trait	Variant type	Chr^a^	Peak SNP^b^	−log_10_(*P*‐value)	Gene count^c^
PH	SNP	1	69 987 248	9.93	4
PH	Indel	4	7 622 167	8.60	8
PH	CNV^d^	7	9 225 709	7.04	1
Tannin	SNP	3	60 368 179	8.27	15
Tannin	SNP	3	60 722 769	9.80	6
Tannin	SNP	8	45 442 123	11.87	2
Tannin	SNP	8	61 186 817	9.92	9

^a^ Chromosome abbreviated as Chr.

^b^ Single‐nucleotide polymorphism abbreviated as SNP.

^c^ Total genes within 20 kb of the peak SNP for the associated locus.

^d^ Copy number variant abbreviated as CNV.

Tannin content is another important domestication trait. While higher tannin content lowers nutrient uptake (Xiong et al., 2019), the presence of such phenolic compounds can conversely be important in reducing pest damage (Wu et al., 2019) and provide antimicrobial (Shields et al., 2021) and antioxidant activities that improve gut health (Xiong et al., 2019). One of the established primary regulators of tannin content is *Tan1*, which was identified in our GWAS for tannin content using all variant types (Figure 5b). Another important locus, *Tan2* (*TT8*: Sobic.002G076600), was not identified in GWAS using tannin content likely due to duplicate recessive epistatic interactions between the *Tan1* and *Tan2* loci (Wu et al., 2019). However, when we conducted GWAS using phenotypic data indicating presence or absence of a testa layer in our SAP accessions using a probit BSLMM, the *Tan2* locus showed a significant association using the SNP markers (Figure S12). Two novel associations with strong peaks were identified for tannin content with SNPs, indels, and CNVs between 60 and 61 Mb of Chr3 (Figure 5b; Figure S13). Previously, a significant association had been identified with 3‐deoxyanthocyanidins around 59.7 Mb of Chr3 (Rhodes et al., 2014). The novel loci at 60–61 Mb of Chr3 consist of several potential candidate genes that are involved in membrane transport, aromatic amino acid synthesis, and terpenoid pathways (Table 2). The top SNP at approximately 60.7 Mb was located within Sobic.003G270500, a gene encoding a farnesyl diphosphate transferase, which functions in the biosynthesis of terpenes and terpenoids (Davis & Croteau, 2000). Similarly, peaks on Chr7 at approximately 61.1 Mb and Chr9 at approximately 53.8 Mb were previously associated with proanthocyanidins (Rhodes et al., 2014), and the peak on Chr8 at approximately 61.1 Mb has been associated with both polyphenol content and grain color (Rhodes et al., 2014).

### Pleiotropy analysis for grain yield and quality traits

Using the 25 traits collected by Boyles et al. (Boyles et al., 2016) and Sapkota et al. (Sapkota, Boyles, et al., 2020), we first performed GWAS for all traits using LMMs in GEMMA (Zhou & Stephens, 2012). From those initial LMM results, 19 were subsequently analyzed for pleiotropic effects using *MashR* (Urbut et al., 2019). *MashR* uses empirical Bayes methods to estimate patterns of similarity among conditions, and the resulting patterns are then used to improve the accuracy of effect estimates. Over 10 000 markers exhibited significant pleiotropic effects – nearly 16× more than was identified for over 100 traits using GBS data – across the sorghum genome with many well‐known loci such as *Dw1*, *Dw2*, *Dw3*, *Ma1*, and *Ma3* (Figure S14) exhibiting strong pleiotropic effects across multiple traits (Mural et al., 2021). Many markers demonstrated an effect across 10 or more traits, and only Chr10 did not exhibit significant pleiotropic effects across more than five traits. Association results for various traits showed strong correlation between each other in pleiotropic analyses of grain yield and quality traits (Figure S15, Figure S16).

### Genome‐wide prediction using WGS and GBS markers

**Figure 6:**
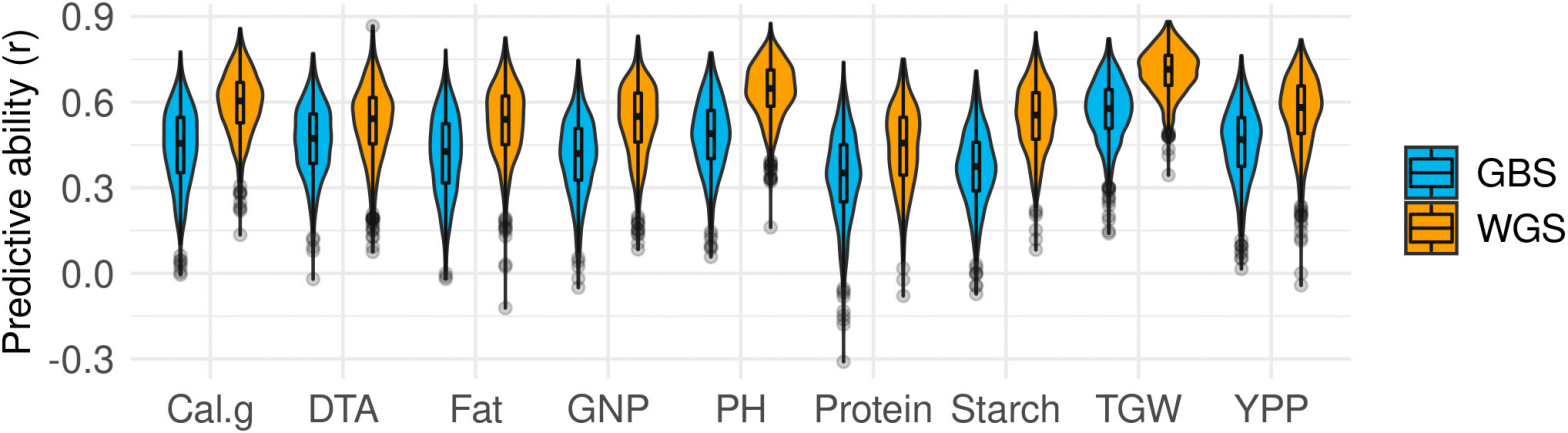
Predictive ability for genomic prediction of traits using whole‐genome sequencing (WGS) and genotyping‐by‐sequencing (GBS) markers. Cal.g, calories per gram; DTA, days to anthesis; GNP, grain number per primary panicle; PH, plant height; TGW, 1000‐grain weight; YPP, yield per primary panicle. [Colour figure can be viewed at wileyonlinelibrary.com]

The value of WGS marker data was further explored by estimating improvements to genomic prediction accuracy compared to GBS markers. Prediction results using WGS marker data showed significantly higher predictive ability (*P* < 2e−16) for all traits compared to GBLUP models using GBS marker data (Figure 6). Predictive ability was, on average, 29% higher for WGS, with increases ranging from 13 to 47% across the traits studied. Mean predictive abilities ranged from 34 to 57% for GBS and from 44 to 71% for WGS, with 1000‐grain weight and protein having the highest and lowest predictive abilities across both marker types, respectively. Among the traits, starch showed the largest (47%) increase in mean predictive ability from GBS to WGS, while days to anthesis showed the smallest (13%) increase.

## DISCUSSION

Since its development, the United States Sorghum Association Panel has served as a pivotal resource for genetic dissection and as a source of genetic diversity for breeding (Boyles et al., 2019; Casa et al., 2008; Xin et al., 2021). The SAP differentiates itself from other sorghum panels (Boatwright et al., 2021; Brenton et al., 2016; Morris et al., 2013) through its composition, which was designed to capture broad phenotypic and genetic diversity across important U.S. breeding lines and converted tropical lines. This is in contrast to panels such as the Bioenergy Association Panel, which was restricted to tall, photoperiod‐sensitive, late‐maturing accessions (Brenton et al., 2016), or any of the multi‐parent populations (Boatwright et al., 2021; Bouchet et al., 2017). To expand the breadth and depth of genomic data available for this crucial diversity panel, we sequenced the accessions in the SAP at a higher depth and provide reliable, high‐density genome‐wide markers for elucidating the genetic architecture of traits and propelling genomics‐assisted breeding. Previously, this panel was only genetically characterized with GBS (Boyles et al., 2016; Morris et al., 2013), which can be biased toward genic sequences and therefore may misrepresent diversity across the population or an individual within the population (Pootakham et al., 2016). Available GBS data captured 268 896 biallelic variants after quality filtering, which is an order of magnitude fewer than what was captured using WGS in this study. Similarly, GBS methods do not capture insertions or deletions with the same fidelity as WGS methods. Here, with corroborating evidence, we demonstrate the inherent value of WGS data in reduced type‐I and type‐II errors, improved mapping resolution by capturing more recombination events, increased depth of variants called, and added benefits of different identifiable variants leading to improvement in genetic dissection and genome‐wide prediction. Further, these broadly diverse data will improve pan‐genomic efforts that use either a single reference genome, as demonstrated here, or multiple, diverse reference genomes by capturing variation at a higher scale than can be easily managed through multi‐reference pan‐genome approaches.

### High‐density variants for genomic research and breeding

Apart from SNP markers, we identified a substantial number of indels and CNVs that contribute to our understanding of the complexity of the sorghum genome and the evolutionary processes that result in or develop from that complexity (Bolger et al., 2014). To date, there are only a few large‐scale studies that have evaluated the utility of high‐throughput indel data for GWAS and genomic prediction in human cohorts while no such study was found in plants (Dai et al., 2020; Say, 2017). Detection of SNPs is significantly easier than indel and CNV identification due to sequencing and reference biases, library preparation requirements, and algorithmic artifacts (Dai et al., 2020; Fang et al., 2014). Indels represent the second‐most common type of genetic variant. Yet, their value for identifying genome‐wide associations has been overlooked due to limitations in both production cost and scalability (Say, 2017). Few studies in sorghum have examined indels and CNVs at scale (Ruperao et al., 2021; Songsomboon et al., 2021; Tao et al., 2021; Zhang et al., 2014; Zheng et al., 2011), and none of these demonstrated the comparative value of all three variant types for GWAS. In fact, comparisons across all three variant types are limited to human studies where funds and scalability are less limiting, but even human studies lack a comprehensive review of the topic (Dai et al., 2020; Say, 2017). Identification of indels and CNVs requires unbiased high‐throughput sequencing or long‐read sequencing to confidently call variants (Abel & Duncavage, 2013), and as the SAP was sequenced at a coverage of approximately 38×, this dataset is uniquely suited to obtain high‐quality variants of all three types. The number of SNPs generated in our study is consistent with the sequencing depth and population scale differences as previously reported for WGS in sorghum (Mace et al., 2013). However, the variants captured here cover more accessions than any other sorghum panel sequenced at similar depths.

### Population structure, haplotypes, and variant graphs

Population structure analyses conducted using SSR markers were confirmed by the development of restriction site‐associated DNA sequencing, such as GBS (Brown et al., 2011; Casa et al., 2008; Mir et al., 2013; Morris et al., 2013). However, the true value of GBS was realized in downstream applications that showed increased mapping resolution for genome‐wide association studies due to increases in genome coverage and ease of genotyping compared to SSR markers (Mir et al., 2013). Similarly, results for population structure and genetic diversity analysis based on WGS data are similar to results based on GBS‐based markers despite increased marker density, which is not surprising considering SSR markers accurately capture population structure despite having much lower coverage than GBS markers (Friel et al., 2021). Consistent with previous characterizations for the SAP, our population structure analysis subdivided the population into approximately six groups, which is consistent with the four botanical racial types, a milo subpopulation that includes the durra‐bicolor of historic importance in breeding and one admixed group that includes some durra race accessions, mixed‐race accessions, and bicolor accessions that are thought to be the early domesticate and do not form a separate cluster (Brown et al., 2011; Harlan & Stemler, 1976; Sapkota, Boyles, et al., 2020; Wang et al., 2013). The average LD decay distance was approximately 20 kb (*r* ^2^ < 0.2) for the whole genome but varied across chromosomes (Hamblin et al., 2004). Notably, Chr6 failed to reach background levels (*r* ^2^ < 0.2), which is consistent with previous results that found limited recombination on Chr6 (Hu et al., 2019; Wang et al., 2013).

To date, there have been three sorghum pan‐genomes published (Jensen et al., 2020; Ruperao et al., 2021; Tao et al., 2021). These pan‐genome construction efforts in sorghum either utilized a smaller population size (N = 176) and lower coverage (approximately 10×) (Jensen et al., 2020; Ruperao et al., 2021) or called variants using sequence differences across multiple references (Tao et al., 2021). Here, we identified more variants using higher coverage across more individuals, which will act as a pivotal resource for future pan‐genomics in sorghum. Pan‐genomes have the potential to provide significantly more information concerning potential haplotype structure across diverse panels than traditional reference genomes and can reduce the effects of reference sequence bias on read mapping and subsequent variant calling (Bayer et al., 2020).

While some pan‐genomic tools utilize pairwise alignment of multiple reference genomes to generate a pan‐genome (Li et al., 2020), the iterative alignment of reference genomes can result in a biased pan‐genome (Hickey et al., 2020). The broad‐scale high‐throughput sequencing of diverse accessions can be foundational for development and visualization of variant graphs (Figure S17), particularly when the degree of large structural variants in a population is low. While variant graphs and pan‐genomics are the future of reference‐based genomics, we identified more quality variants using the GATK and Hecaton than utilizing a variant graph approach with *vg*. Thus, while construction of a variant graph has the potential to reduce reference bias, the gains in bias reduction should be measured against the potential variant coverage that established variant callers can provide. To fully exploit the benefits of variant graphs, variants should be called using pan‐genomes constructed from multiple references so that contrasting haplotypes within a population or species can be captured. Such future projects can also generate advanced pan‐genomes with variant data from diversity panels such as those described here to capture a broader range of the genetic variation represented in sorghum.

### Distinct signatures for historic selection versus recent selection

Since the accessions in the SAP include various botanical races arising from evolutionary divergence and local adaptation, the genetic differentiation between racial types is indicative of differences arising from historic selection during domestication. Additionally, the accessions can also be divided differently into two groups: one group of individuals in the SAP are the converted lines from the SCP that were introgressed with maturity and height loci for photoperiod conversion and short stature (Klein et al., 2008; Stephens et al., 1967), whereas the other group of individuals include the cultivars that were not only temperate‐adapted but were bred and selected through multiple generations and as a result new recombination events have resulted in potentially different allelic combinations across the genome (Klein et al., 2008; Sapkota, Boyles, et al., 2020).

The genome‐wide signatures of selection were distinct for historic selection during domestication and local adaptation compared to selection signatures resulting from recent selection activities during photoperiod conversion and/or breeding (Figure 4). Since the converted lines were distributed across all botanical races, there were no distinct F_st_ peaks around the three genomic regions in Chr6, Chr7, and Chr9 that harbor the introgression for dwarfing and maturity genes into exotic tropical lines by the SCP. Thurber et al. (Thurber et al., 2013) had previously shown that introgression during the conversion process did not have any bearing on population structure analysis of converted lines. Based on the F_st_ peaks observed between the converted accessions and temperate‐adapted breeding lines, the haplotypes for the introgressed region in Chr6 could be distinct from those in converted lines, whereas the other two introgressed regions in Chr7 and Chr9 show little differentiation from the breeding lines. This might be due to abundance of recessive alleles for *Dw1* and *Dw3* while the dwarfing allele for *Dw2* locus is rare among the breeding lines.

The genomic region in Chr2 (45–54 Mb) that showed common F_st_ peaks across all races exhibits a combination of positive selection at the beginning of the region with some bottleneck toward the end. The region at 53–54 Mb shows a strong bottleneck below two standard deviations of the mean Tajima's D estimate, and an associated peak for anthracnose resistance was previously identified within this region, around 53.79 Mb of Chr2. This locus contains a large (32 kb) sorghum gene, Sobic.002G169633, which is located approximately 130 kb upstream of the associated GWAS peak and encodes a protein kinase with NB‐ARC and LRR domains (Cuevas & Prom, 2020). The presence of 24 candidate genes with coiled‐coil domains suggests that this region might have several mutations that have been independently, positively selected for across sorghum races during local adaptation. This region of the genome needs to be studied further because it shows strong selection and is important for genetic dissection and breeding for biotic resistance.

Another region that showed signs of a selective sweep between converted and bred lines as well as within the caudatum subpopulation was the region around the *Tan1* locus. The tannin loci have historically been subjected to bidirectional selection because of varied local herbivore threats and human taste sensitivity, resulting in natural variation around these loci across sorghum germplasm (Wu et al., 2019). Caudatum accessions make up 45% of the SAP accessions that were reported to have a pigmented testa layer, whereas 32% of the remaining accessions with pigmented testa were from the mixed subpopulation which also has several accessions closely related to caudatum (File S1). Also, 73% of accessions with pigmented testa were from the converted group, whereas only 12% of accessions in the bred group were pigmented. This difference could result in large allele frequency differences between the two groups around this region (File S1).

### WGS markers improved genome‐wide association and prediction over GBS markers

In addition to the common height loci (*Dw1*–*Dw3*) and tannin content loci (*Tan1*) identified previously using GBS markers, we identified novel associations across two loci for PH and four loci for tannin content that were not identified with GBS markers. Previous studies have demonstrated that WGS improves both the mapping resolution and the ability to identify novel associations over marker data derived from GBS (Höglund et al., 2019). Apart from the three well‐characterized height loci (*Dw1*, *Dw2*, and *Dw3*), we also detected a significant association for the putative *Dw4* locus at approximately 6.6 Mb of Chr6 as previously reported (Miao et al., 2020; Morris et al., 2013). A novel height association detected in Chr1 was located within 1 Mb of a previously reported PH QTL (Marla et al., 2019). While associations at major loci for height overlapped for different variant types, the significant associations for PH on Chr4 and Chr7 that were unique to indel data show that indel variants can overcome limitations of SNP data in detecting potential false negative associations. The tannin locus (*Tan1*) we have identified is consistent with previous association results in the SAP (Rhodes et al., 2014). While the *Tan2* locus was undetected in our tannin content association, as in previous association analyses, we show that probit GWAS using a BSLMM for presence or absence of a testa layer can detect both *Tan1* and *Tan2* loci. As both PH and tannin content represent important phenotypes in sorghum breeding, the consistency in association results compared to previous studies provides validity of this newly developed genomic resource while novel associations show incremental advantage in genetic dissection.

We performed genomic prediction (GBLUP) using a genomic relatedness matrix derived from GBS or WGS markers to compare their predictive ability for agronomic, yield, and quality traits. On average, a 29% increase in predictive ability was observed across nine traits, which is a substantial increase for most of these traits as they are quantitative and complex traits. This improvement in predictive ability is due to the ability of WGS markers to capture additive genomic relationships better because of increased density and coverage of genetic markers, subsequently resulting in improvement in the total genetic variance explained by the model (Heffner et al., 2009). And since genetic gain is directly proportional to selection accuracy, such improvements in accuracy of prediction will have a cumulatively positive effect on the long‐term genetic gain across sorghum breeding programs (Faville et al., 2018). Thus, these WGS variants will provide both immediate improvements, via improved associations and predictive ability, and long‐term improvements, via the compounding effects of genetic gain.

## CONCLUSION

Approximately 44 million variants of diverse types were called using the WGS data for the SAP, and these data represent a major increase in the density and variant types available for future sorghum studies. These data further open opportunities for detailed variant graphs, improved genomic prediction, and detection of novel loci facilitating sorghum improvement as we have demonstrated here. As pan‐genomic methods continue to develop, sources of broad genotypic diversity will be pivotal to capturing diverse haplotypes vital to crop improvement, but these data also provide immediate improvement to genomic selection and the identification of genotype‐to‐phenotype associations. Variant data are provided as a community resource for the continued development of this multi‐purpose, climate‐resilient crop.

## AUTHOR CONTRIBUTIONS

JLB and SS conceptualized, developed, and implemented the study design and wrote the manuscript. JLB performed sequence analyses, variant data generation, and association analyses. SS performed population genomic analyses and genomic prediction. HJ performed sequence quality control analysis. SK, JCS, RB, and ZB acquired funding, provided supervision, and managed the project. All authors reviewed the manuscript and approved the manuscript for publication.

## CONFLICT OF INTEREST

JCS has equity interests in Data2Bio LLC, a company that provides genotyping services using sequencing technology. The authors declare no other conflicts of interest.

## Supporting information


**File S1.** Accessions in the sorghum association panel along with metadata for population clusters, origin, racial classification, and testa pigmentation.

**Supporting Information S1** Supporting information is available at Figshare through *The Plant Journal*'s supplemental link.
**Table S1.** GATK variants and corresponding types.
**Table S2.** Haplotype blocks per sorghum chromosome.
**Figure S1.** Histograms of read coverage per sample.
**Figure S2.** Cumulative genome coverage across samples.
**Figure S3.** Guanine‐cytosine content distribution across samples.
**Figure S4.** Total count of nucleotide substitutions across sorghum chromosomes.
**Figure S5.** Indel length distribution.
**Figure S6.** Linkage disequilibrium decay across each sorghum chromosome and across the genome.
**Figure S7.** Cumulative variance explained across the SAP principal components.
**Figure S8.** Discriminant analysis of principal components across varying values of k (number of clusters).
**Figure S9.** Heatmap showing genomic relatedness between individual accessions within the sorghum association panel.
**Figure S10.** Regions across the sorghum genome demonstrating selective sweeps for various subpopulations based on ADMIXTURE analysis.
**Figure S11.** Measures of Tajima's D across the genome within the photoperiod converted lines (Conv), breeding lines (Bred), and the whole population.
**Figure S12.** Genome‐wide association for presence of testa layer using a probit Bayesian sparse linear mixed model.
**Figure S13.** Heatmap for linkage disequilibrium around association peak for tannin content in chromosome 3.
**Figure S14.** Genome‐wide measures for pleiotropic effects of associated regions for 19 traits.
**Figure S15.** Trait correlation across the sorghum genome for the 19 traits in the pleiotropy analysis.
**Figure S16.** Genome‐wide associations for grain yield components (a) and grain composition (b) using linear mixed models.
**Figure S17.** Variant graph of Dw1 locus at different aspects demonstrating macro‐ and micro‐variations in the locus structure.

## Data Availability

Raw WGS data are available at the European Nucleotide Archive under accession PRJEB50066 (ERP134618). Variant calls are available through the European Variant Archive under accession PRJEB51985 or upon request. Scripts are available on GitHub (https://github.com/jlboat/SAP).
