# Supplementary material for: Sorghum Association Panel whole‐genome sequencing establishes cornerstone resource for dissecting genomic diversity
Source: Plant J. 2022 Jul 5;111(3):888–904. doi: 10.1111/tpj.15853 (PMC9544330; doi:10.1111/tpj.15853)
Supplement: Supplementary file 2 — Supporting Information S1 Supporting information is available at Figshare through The Plant Journal's supplemental link. Table S1. GATK variants and corresponding types. Table S2. Haplotype blocks per sorghum chromosome. Figure S1. Histograms of read coverage per sample. Figure S2. Cumulative genome coverage across samples. Figure S3. Guanine‐cytosine content distribution across samples. Figure S4. Total count of nucleotide substitutions across sorghum chromosomes. Figure S5. Indel length distribution. Figure S6. Linkage disequilibrium decay across each sorghum chromosome and across the genome. Figure S7. Cumulative variance explained across the SAP principal components. Figure S8. Discriminant analysis of principal components across varying values of k (number of clusters). Figure S9. Heatmap showing genomic relatedness between individual accessions within the sorghum association panel. Figure S10. Regions across the sorghum genome demonstrating selective sweeps for various subpopulations based on ADMIXTURE analysis. Figure S11. Measures of Tajima's D across the genome within the photoperiod converted lines (Conv), breeding lines (Bred), and the whole population. Figure S12. Genome‐wide association for presence of testa layer using a probit Bayesian sparse linear mixed model. Figure S13. Heatmap for linkage disequilibrium around association peak for tannin content in chromosome 3. Figure S14. Genome‐wide measures for pleiotropic effects of associated regions for 19 traits. Figure S15. Trait correlation across the sorghum genome for the 19 traits in the pleiotropy analysis. Figure S16. Genome‐wide associations for grain yield components (a) and grain composition (b) using linear mixed models. Figure S17. Variant graph of Dw1 locus at different aspects demonstrating macro‐ and micro‐variations in the locus structure. [file TPJ-111-888-s001.pdf]

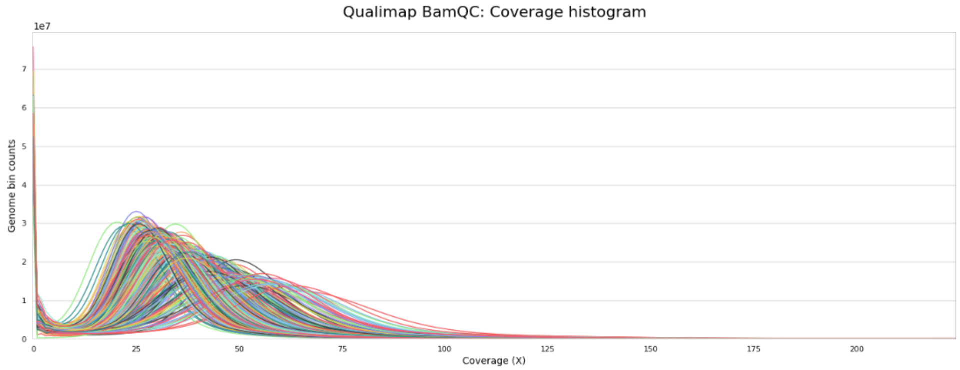

S1 Fig. Histograms of read coverage per sample. The peak at zero coverage may indicate regional differences across the genome due to sequences absent from the given sample (due to a deletion or structural rearrangement), present in the sample but not successfully sequenced (due to bias in sequencing or preparation), or sequenced but not successfully mapped to the reference (due to the choice of mapping algorithm, the presence of repeat sequences, or mismatches caused by variants or sequencing errors).

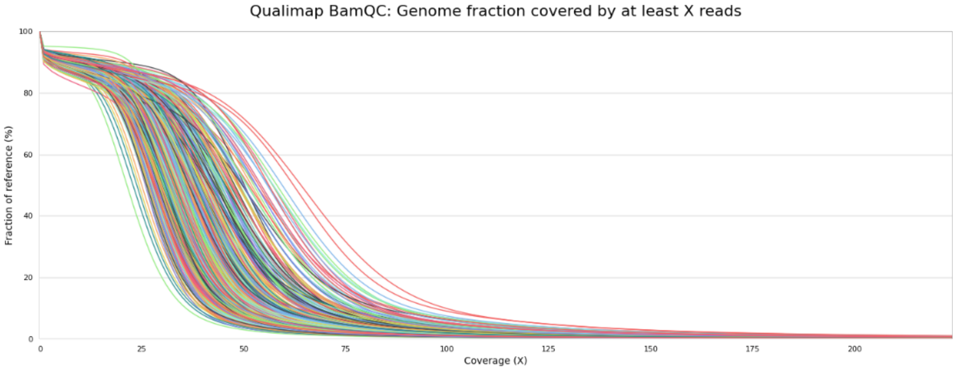

S2 Fig. Cumulative genome coverage across samples. Figure indicates the fraction of the reference genome with at least the given depth of coverage.

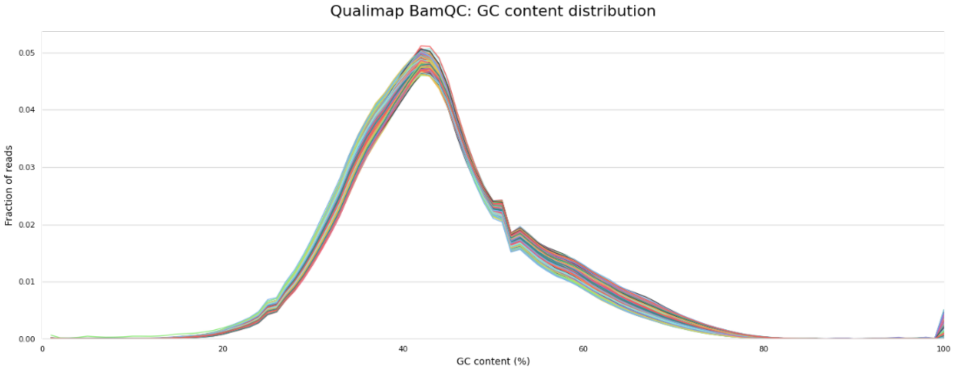

S3 Fig. Guanine-Cytosine content distribution across samples. Each solid line represents the distribution of GC content of mapped reads for a given sample.

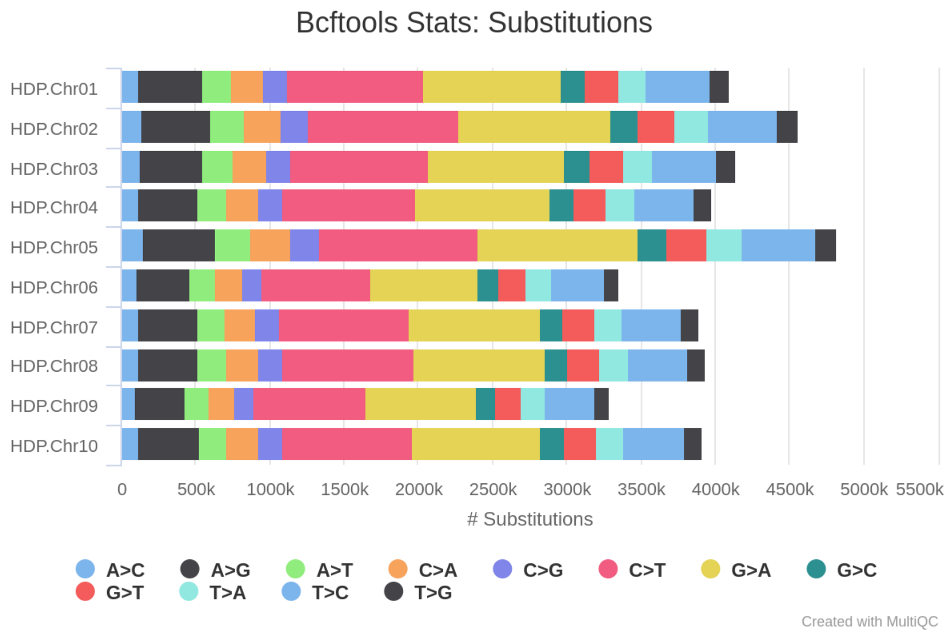

S4 Fig. Total count of nucleotide substitutions across sorghum chromosomes.

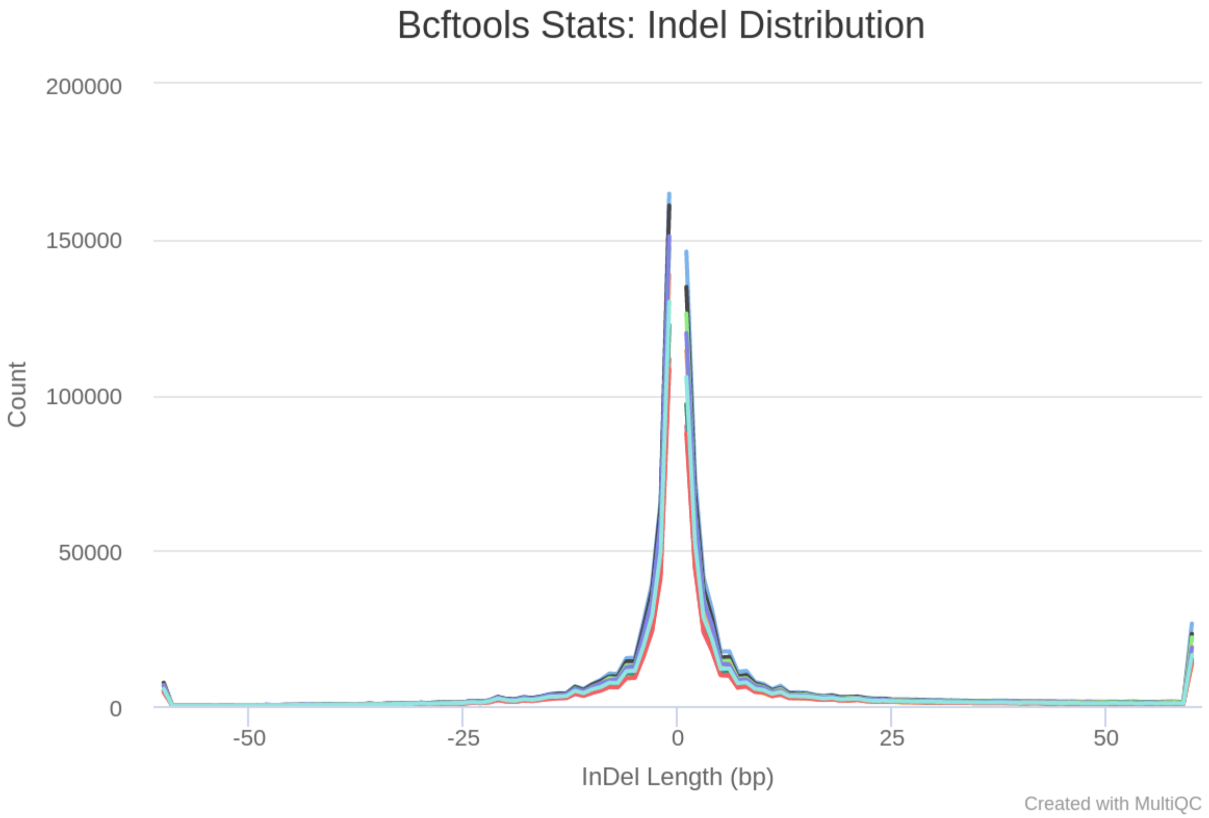

S5 Fig. Indel length distribution. Each solid line represents the distribution of indel length for a given sample with positive values indicating an insertion and negative values indicating a deletion.

S1 Table. GATK variants and corresponding types.

Sample Name	Vars	SNP	Indel	Ts/Tv
**Chr01**	4,628,321	3,967,265	736,554	1.96
**Chr02**	5,037,850	4,414,730	697,485	1.87
**Chr03**	4,574,808	4,006,134	634,931	1.88
**Chr04**	4,369,055	3,844,884	589,362	1.91
**Chr05**	5,224,039	4,658,864	641,858	1.83
**Chr06**	3,661,089	3,248,146	461,694	1.83
**Chr07**	4,205,082	3,766,034	493,073	1.91
**Chr08**	4,260,533	3,805,883	514,874	1.87
**Chr09**	3,587,831	3,185,584	451,220	1.94
**Chr10**	4,263,179	3,779,126	545,768	1.88

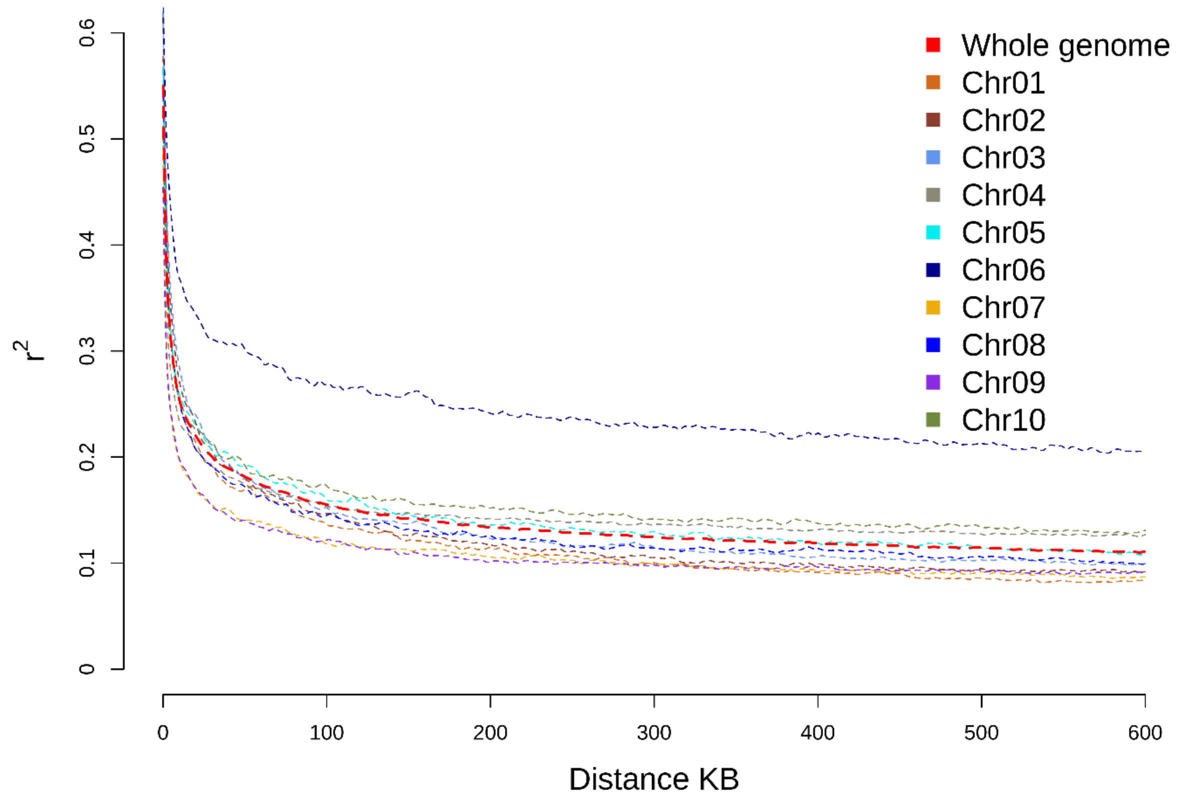

S6 Fig. Linkage disequilibrium decay across each sorghum chromosome and across the genome.

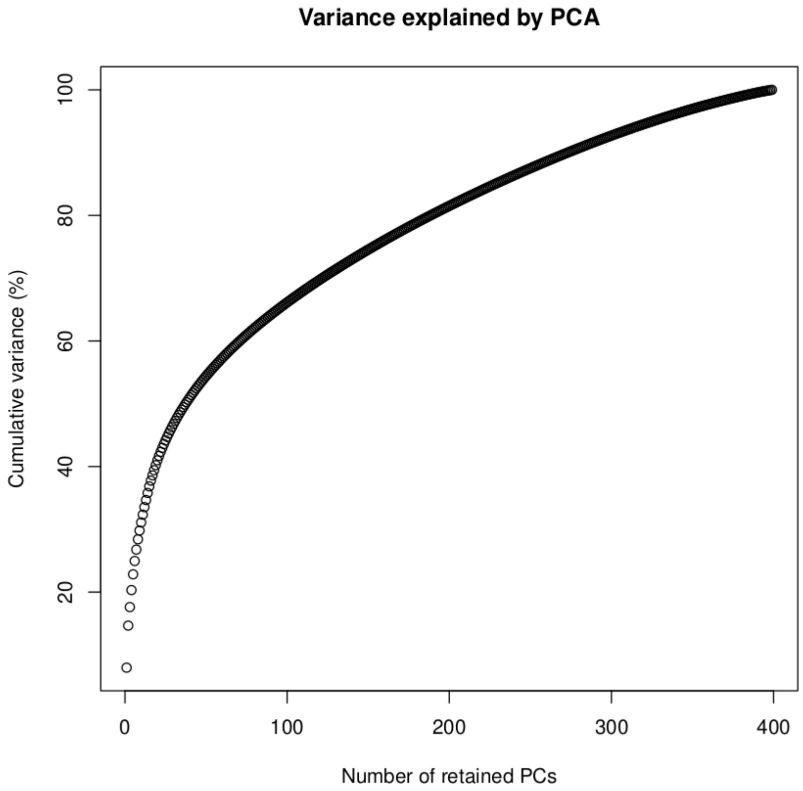

S7 Fig. Cumulative variance explained across the SAP principal components.

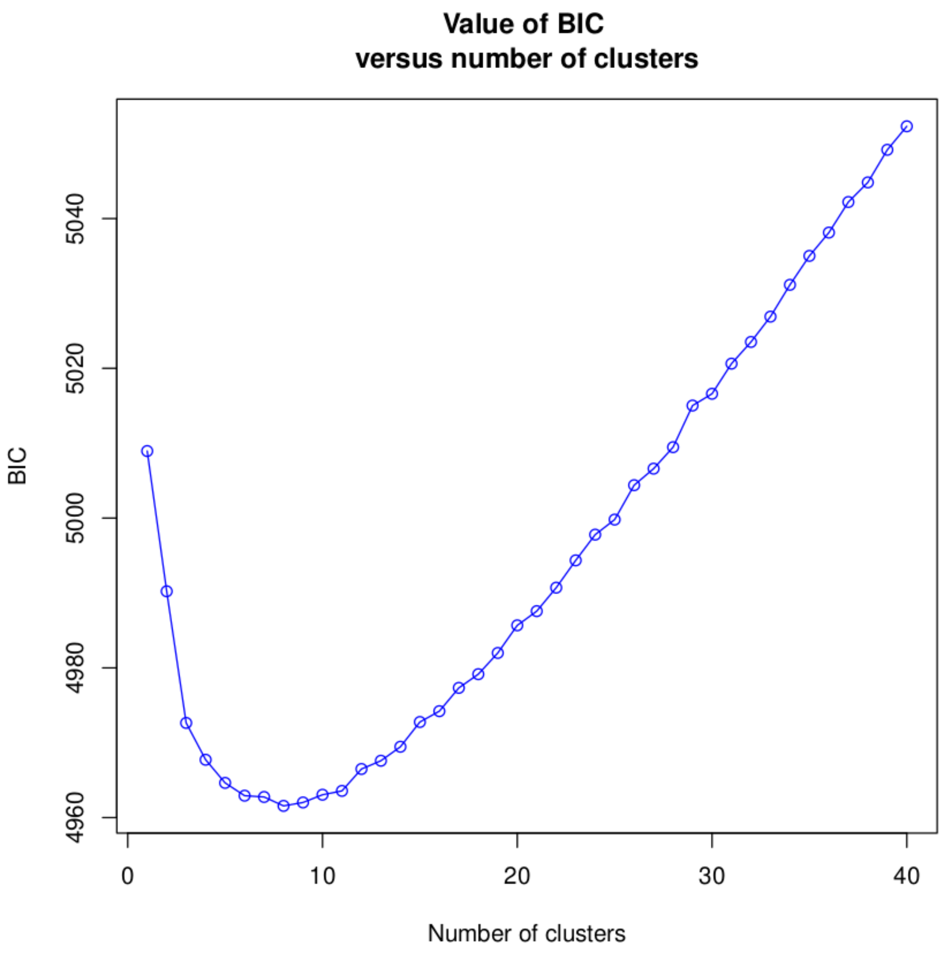

S8 Fig. Discriminant analysis of principal components across varying values of k (number of clusters).

S2 Table. Haplotype blocks per sorghum chromosome.

Chromosome	Haplotype Block Count
Chr01	3,906
Chr02	4,196
Chr03	3,838
Chr04	4,183
Chr05	4,312
Chr06	2,818
Chr07	2,089
Chr08	3,420
Chr09	2,637
Chr10	3,630

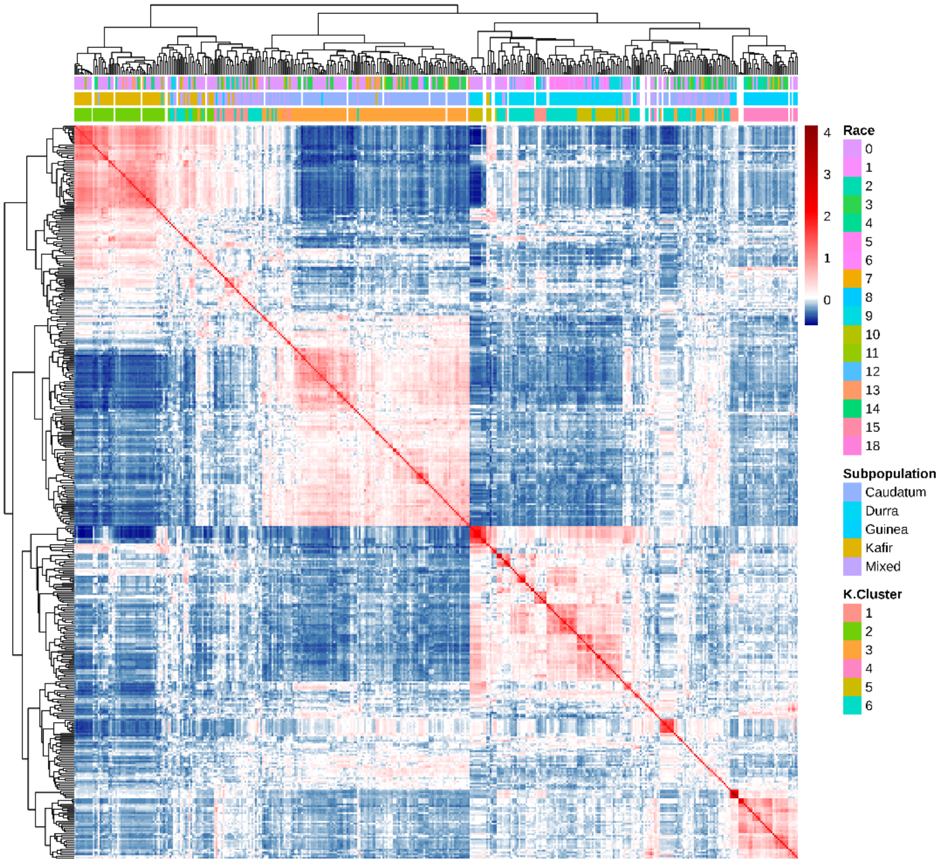

S9 Fig. Heatmap showing genomic relatedness between individual accessions within the sorghum association panel. Sorghum relatedness matrix was generated using the Van Raden (2008) method. K clusters represent the group designation derived from admixture analysis.

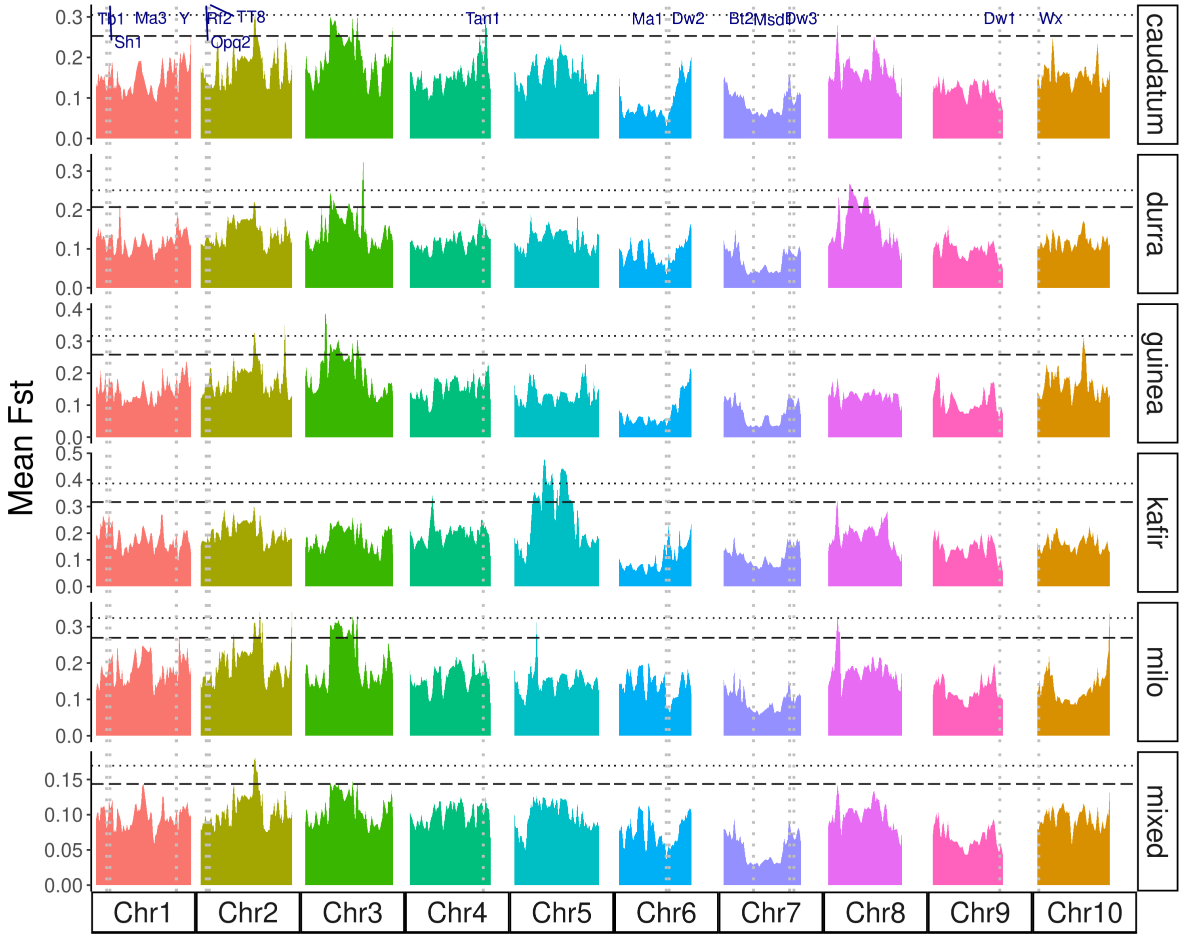

S10 Fig. Regions across the sorghum genome demonstrating selective sweeps for various subpopulations from ADMIXTURE analysis. Horizontal dashed and dotted lines represent two and three standard deviations from the mean F_{st}, respectively. Vertical lines show genomic position of the genes/loci shown in the top panel.

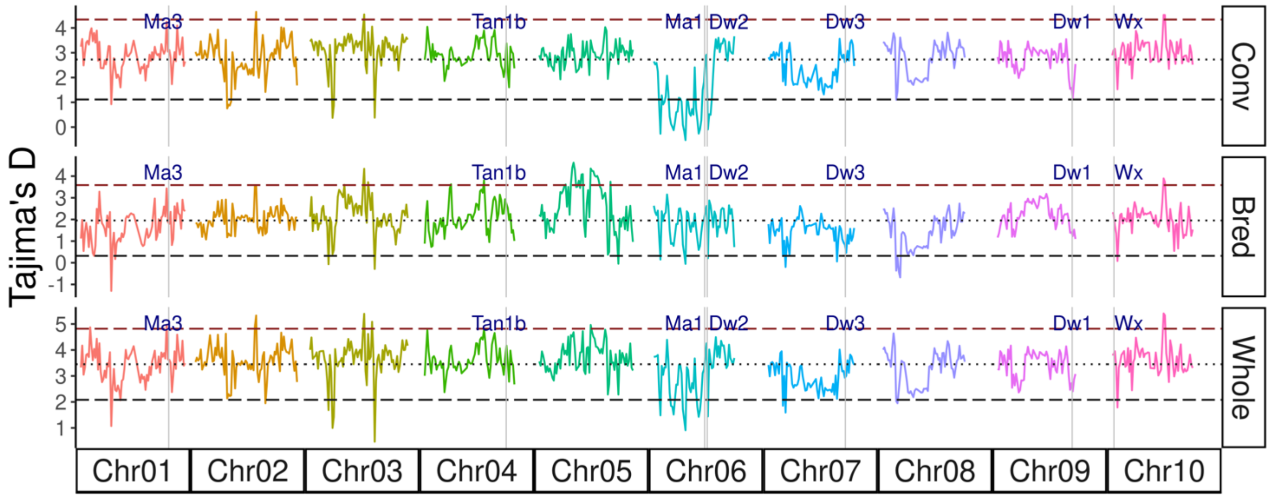

S11 Fig. Measures of Tajima's D across the genome within the photoperiod converted lines (Conv), breeding lines (Bred), and the whole population. Dotted lines show mean Tajima's D for each group and dashed lines show two standard deviations from the mean estimates. Vertical gray lines show genomic position of known height (Dw), maturity (Ma), or tannin (Tan) loci.

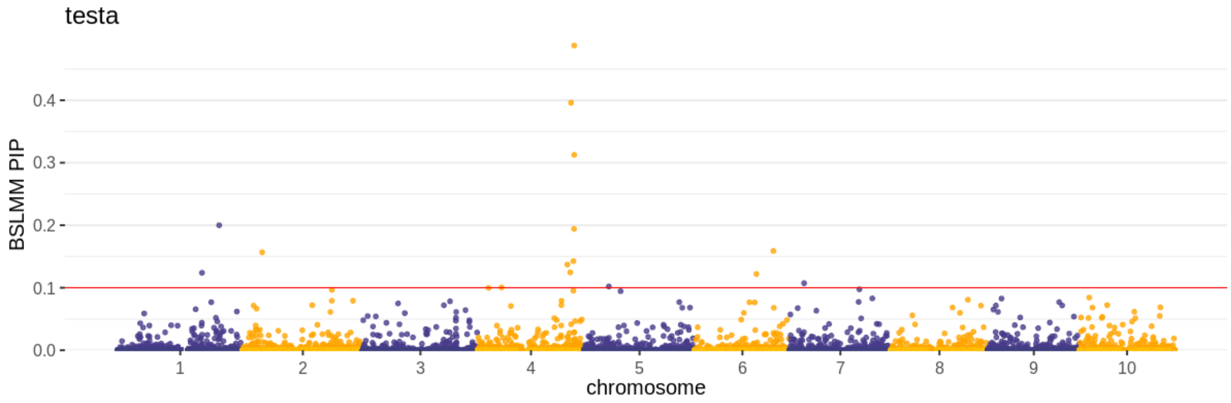

S12 Fig. Genome-wide association for presence of testa layer using probit Bayesian sparse linear mixed model (BSLMM). PIP: posterior inclusion probability.

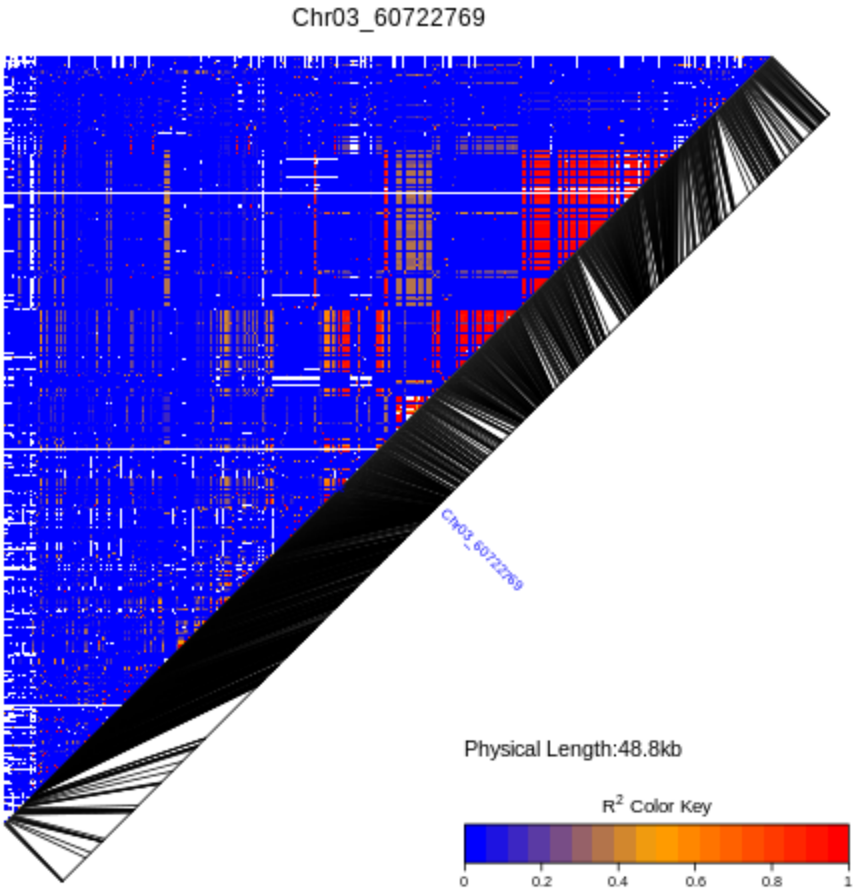

S13 Fig. Heatmap of linkage disequilibrium around association peak for tannin content in chromosome 3.

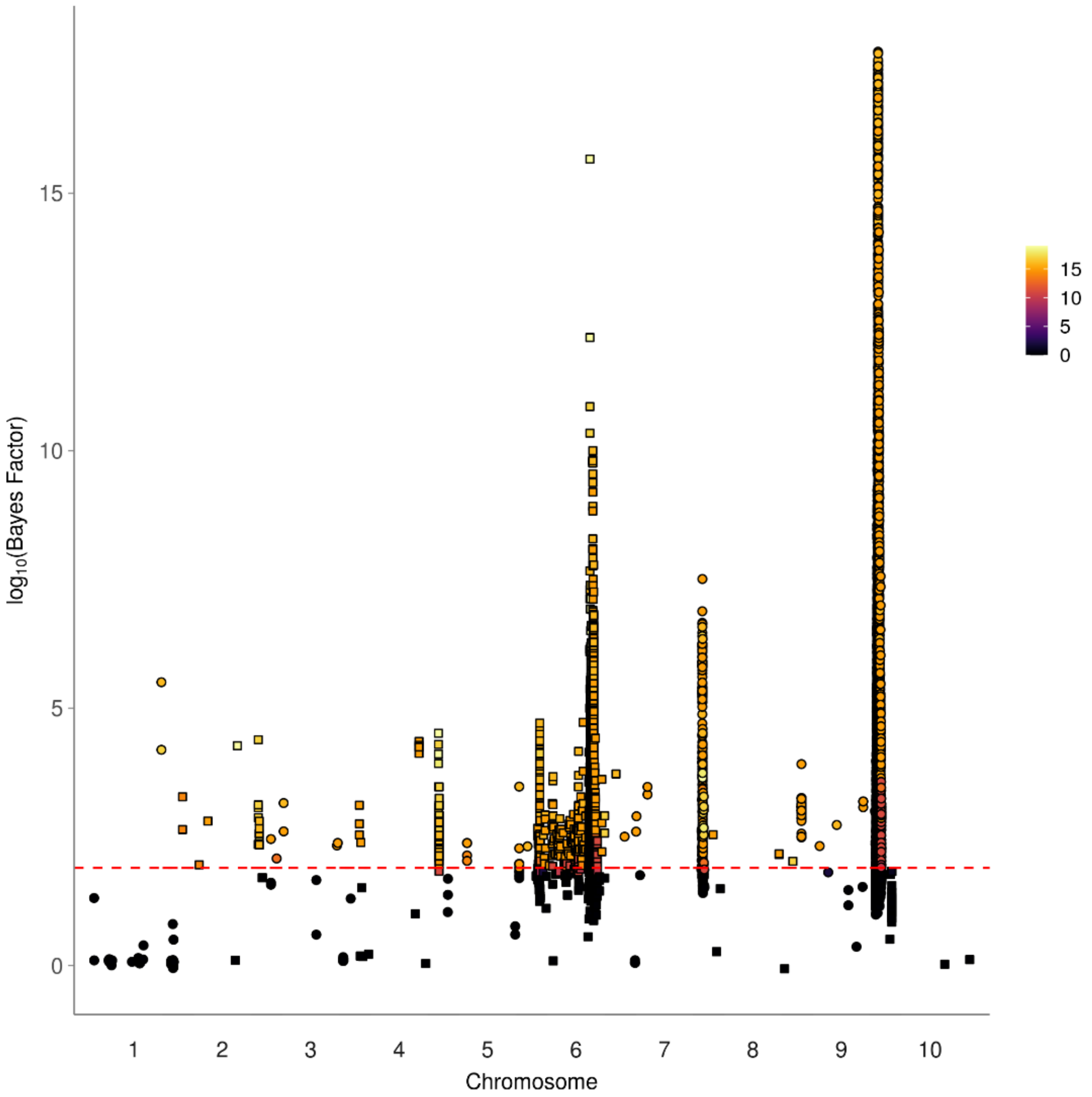

S14 Fig. Genome-wide measures of pleiotropic effects of associated regions for 19 grain yield and quality traits.

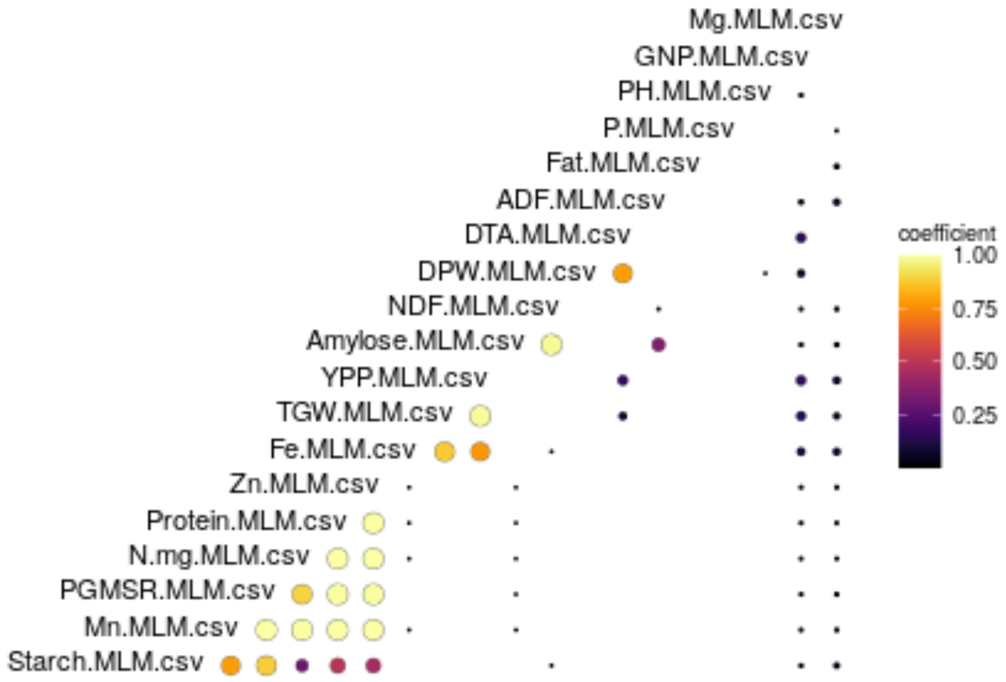

S15 Fig. Trait correlation across the sorghum genome for the 19 traits in the pleiotropy analysis.

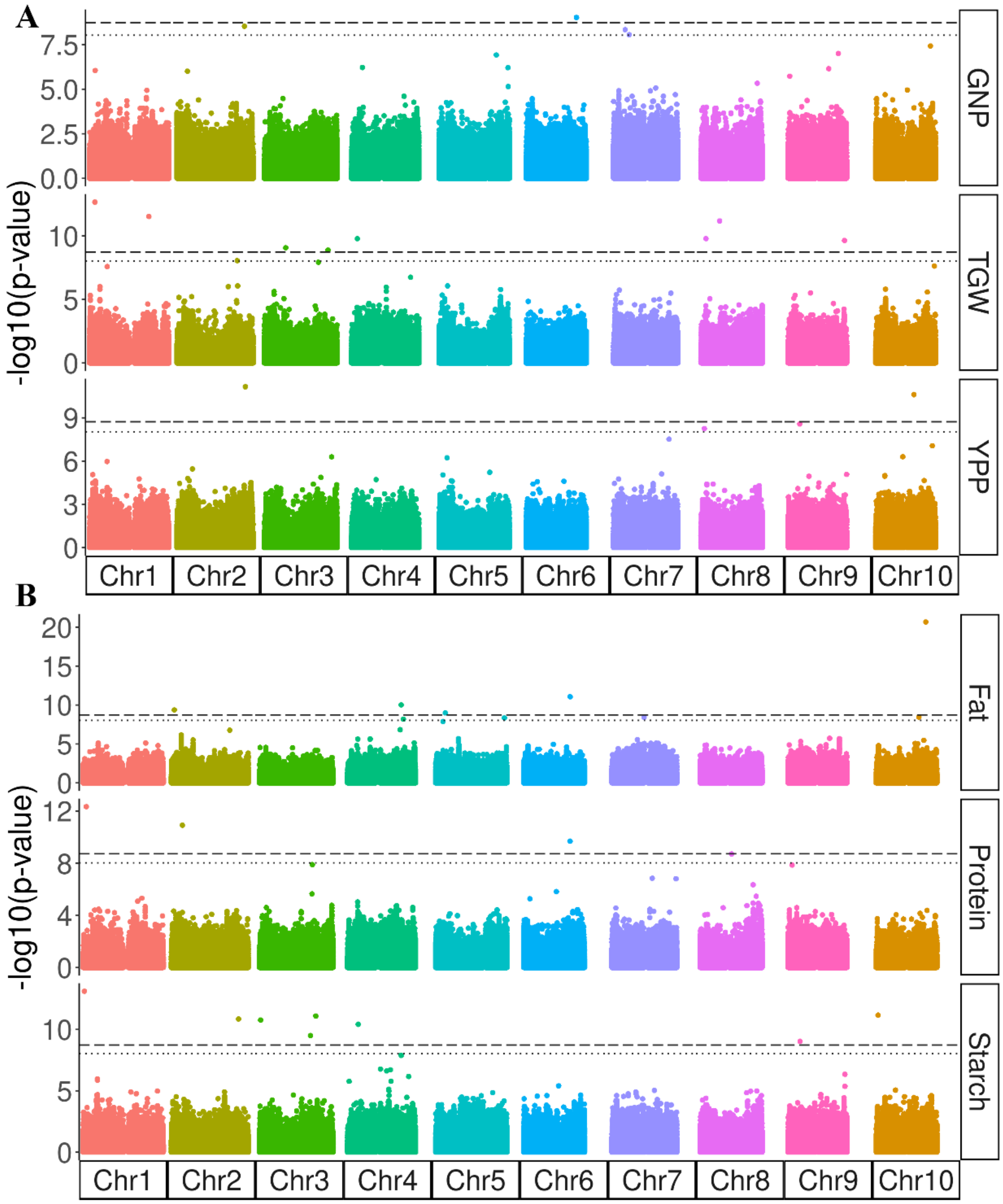

S16 Fig. Genome-wide associations for grain yield components (A) and grain composition (B). Grain number per panicle (GNP), thousand-grain weight (TGW), and yield per panicle (YPP). Horizontal dotted lines represent a Bonferroni-adjusted threshold of 0.05, and the horizontal dashed lines represent a Bonferroni-adjusted threshold of 0.01.

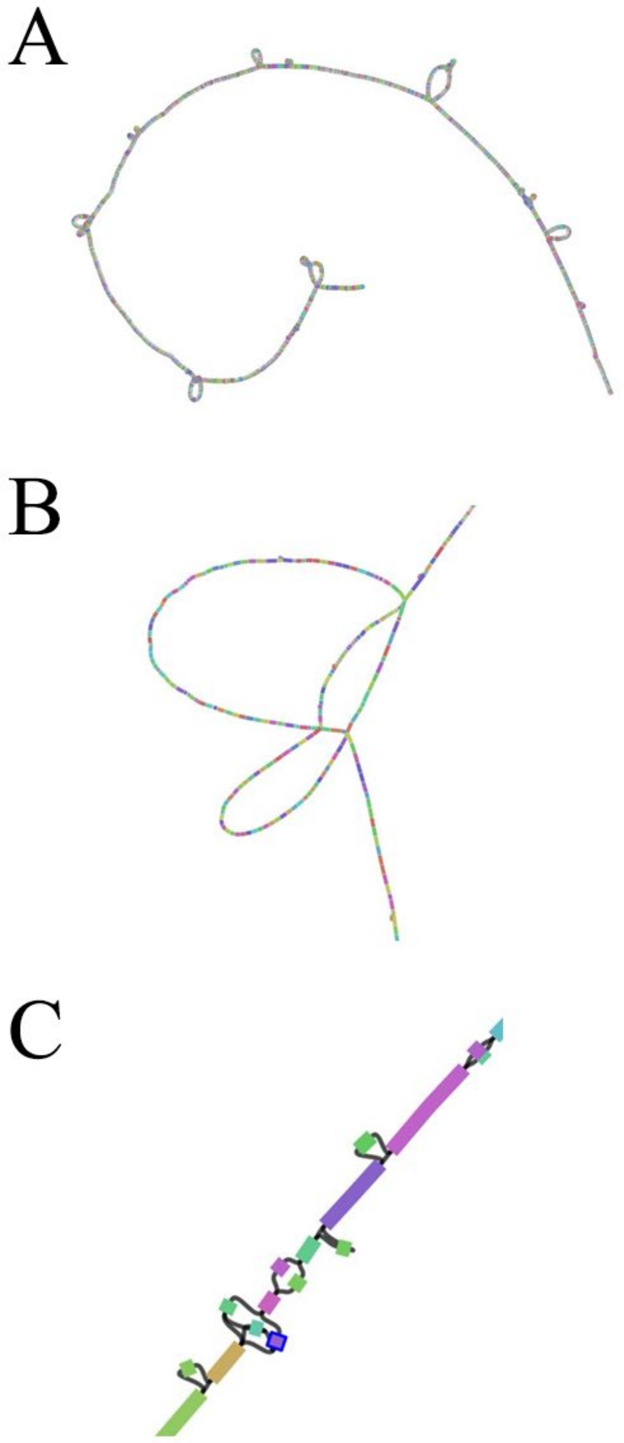

S17 Fig. Variant graph of Dw1 locus at different aspects demonstrating macro- and micro-variations in the locus structure. (A) View of the pan-genome at the locus level, which captures all variant types. (B) View of the left-most knot in part (A), which captures the complex structure of insertions and deletions. (C) View of single-nucleotide polymorphisms capture by the pan-genome, which highlights the complex variation at even the most minor scale.
